# The Use of Click-Type Reactions in the Preparation of Thermosets

**DOI:** 10.3390/polym12051084

**Published:** 2020-05-09

**Authors:** Osman Konuray, Xavier Fernández-Francos, Silvia De la Flor, Xavier Ramis, Àngels Serra

**Affiliations:** 1 Thermodynamics Laboratory, ETSEIB Universitat Politècnica de Catalunya, Av. Diagonal 647, 08028 Barcelona, Spain; osman.konuray@mmt.upc.edu (O.K.); xavier.fernandez@upc.edu (X.F.-F.); ramis@mmt.upc.edu (X.R.); 2 Department of Mechanical Engineering, Universitat Rovira i Virgili, Av. Països Catalans 26, 43007 Tarragona, Spain; silvia.delaflor@urv.cat; 3 Department of Analytical and Organic Chemistry, University Rovira i Virgili, c/ Marcel·lí Domingo 1, 43007 Tarragona, Spain

**Keywords:** click-reaction, thermosets, thiol chemistry, azide-yne, Michael addition, Diels-Alder

## Abstract

Click chemistry has emerged as an effective polymerization method to obtain thermosets with enhanced properties for advanced applications. In this article, commonly used click reactions have been reviewed, highlighting their advantages in obtaining homogeneous polymer networks. The basic concepts necessary to understand network formation via click reactions, together with their main characteristics, are explained comprehensively. Some of the advanced applications of thermosets obtained by this methodology are also reviewed.

## 1. Introduction

The development of novel thermosets with requirements of high mechanical and thermal performance for new technologies has moved researchers to incorporate new chemical processes into the preparation of these polymeric materials. Considering the concept proposed by Sharpless [1], click reactions have been utilized over the last few decades as polymerization systems to form new thermosetting materials with enhanced characteristics to meet the demands of the industry. This is because these reactions are fast, can be performed in an air atmosphere, can be triggered either thermally or photochemically, have high yields without side-reactions, and are regiospecific and orthogonal, i.e., two or more different reactions can be combined without interference. For all these reasons, click reactions have been extensively used in several research areas related to polymer science, like in the preparation of dendrimers and other branched structures and in the modification of polymeric surfaces and nanostructures [2,3,4,5,6,7,8]. Clickable groups placed at the chain end or along the backbone provide reactive sites for postpolymerization functionalization. In addition, click reactions can be used to synthesize new monomers or macromonomers [9]. However, in this review, our attention will be centered on the use of click-type reactions in three-dimensional polymerization to prepare thermosets, highlighting the advantages of these chemical processes in the synthesis of crosslinked polymers, their processability and some special applications.

**Scheme 1:**
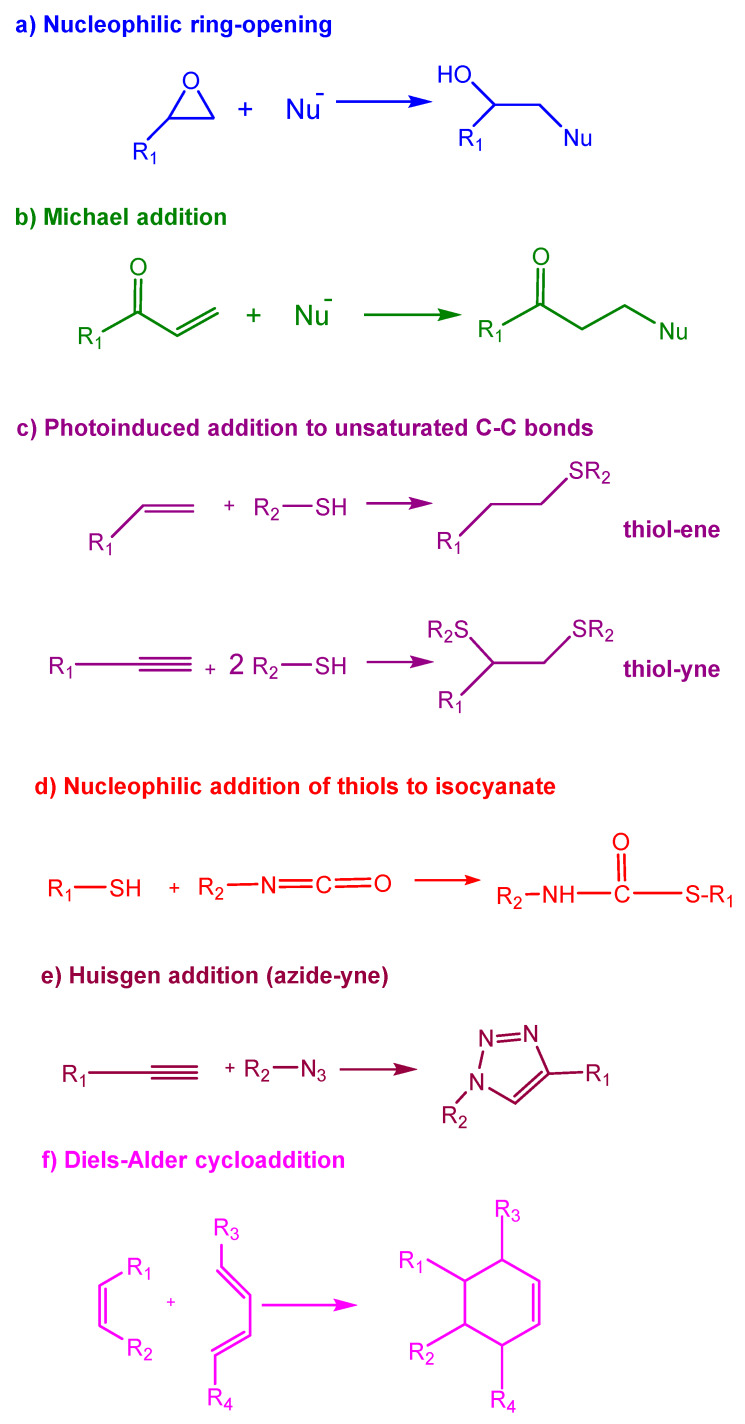
Main click-type reactions used in the preparation of thermosets.

The most notable click reactions that have been previously employed in published studies are depicted in Scheme 1.

The crosslinking process in these reactions follows a polycondensation or step-growth mechanism, which is advantageous in comparison to polyaddition or ring-opening mechanisms. One advantage is the homogeneity of the material. The network is formed in consecutive steps starting from the initial formulation; then, branched structures are generated in the entire polymer matrix with reactive groups as chain ends, until gelation is reached. The polymerization continues until full crosslinking is achieved as long as no vitrification occurs. The homogeneity of the material is enhanced because of the high yield of these reactions which occur without side-reactions or the formation of byproducts.

**Figure 1:**
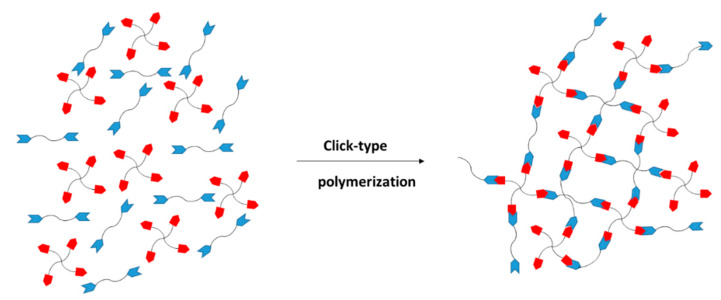
Schematic representation of the crosslinking process using click reactions.

The monomers that react are of type $A_{f}$ and $B_{g}$. The functional group A reacts exclusively with functional group B. The subscripts f and g represent the number of new bonds that can be formed from each monomer in the polymerization reaction. For linear polymers, f and g should be 2, but to produce a network, a higher functionality is needed, and either f or g has to be higher than 2. A representation of the network formation by this methodology is given in Figure 1.

The step-growth characteristic of this polymerization makes it easy to predict the conversion at gelation and the network parameters [10]. It is necessary to keep in mind that these reactions occur in air, which is convenient for technological applications such as coatings. Thus, there are no special requirements, and the polymerization can be performed by heating or irradiation, depending on the reaction. The fact that the reactions occur efficiently with high yields without the cooccurrence of side-reactions leads to an accuracy in the stoichiometry of the formulations which makes it possible to establish complex fabrication processes based on sequential dual curing [11]. Last but not the least, these polycondensation processes do not yield small molecules as other polycondensation reactions do (i.e., water in the polyesterification from acids and alcohols). In the preparation of thermosets, the formation of small molecules like H_2_O, HCl, etc. leads to the formation of bubbles, cavities and irregularities that deteriorate the appearance and properties of the material. For this reason, the use of click reactions to form thermosets is highly advantageous. It is no surprise that epoxy resins are usually cured by amines, which involves a nucleophilic ring-opening reaction; this is the oldest and the most widely used procedure to cure epoxy resins.

## 2. Phenomenological and Theoretical Treatment of Click Reactions in the Preparation of Thermosets

The curing of thermosets is a complex process from the structure, kinetics and properties viewpoints [10,12,13]. As illustrated in Figure 1, a reacting system can evolve from a mixture of unreacted monomers to a fully cured network. However, the extent to which such a transformation can take place, and the network structure (if any) or molecular architecture obtained, depend largely on the time/temperature conditions of the process and on the feed ratio of the monomers. As mentioned, we need to include in the reaction mixture monomers or crosslinking agents with a functionality equal to or higher than three if we want to produce a crosslinked network. However, we must ensure that we employ a sufficient amount so that we end up with a fully crosslinked network when all functional groups are reacted (i.e., under stoichiometric reaction conditions). Alternatively, we can leave a controlled amount of unreacted groups on purpose to get a partially crosslinked material with a tailored structure that can be subject to a postfunctionalization process or a second polymerization reaction that is triggered under controlled conditions [11]. Finally, we can choose to use a feed ratio and functionality of monomers with the intention of producing soluble, high molecular weight and reactive oligomers that will be later mixed with other reagents in order to get a more controlled crosslinking process.

From a structural point of view, the system evolves from a viscoelastic liquid mixture of monomers to a more or less densely crosslinked network. A critical point during this process is gelation, which corresponds to the incipient formation of a 3-dimensional network. Precise knowledge of conversion and time at gelation is essential from a processing point of view, since, at gelation, the bulk of the reacting system starts to become insoluble and ceases to flow. The evolution of the molecular and network architecture during processing will have a deep impact on the rheological and mechanical properties during processing. In consequence, the structure and network build-up process need to be characterized experimentally, but relevant knowledge can also be obtained from a theoretical analysis using suitable mean-field methods.

The molecular and network build-up process is accompanied by an increase in the glass transition temperature of the system, $T_{g}$. This reflects a reduction in mobility due to (a) the decrease in chain-ends caused by the formation of covalent bonds during the reaction, and (b) the reduction in configurational entropy caused by the growing presence of crosslinks in the network structure [14]. This will also have important consequences in terms of reaction kinetics and the achievable extent of the reaction. If the $T_{g}$ of the reactive system increases above the curing temperature, vitrification takes place and the reaction starts to become diffusion controlled instead of chemically controlled [10], slowing down the curing rate significantly and eventually halting the curing process, leading to an incomplete reaction. It is generally accepted that no reaction will take place below the initial glass transition of the monomer mixture, $T_{g0}$, and that $T_{cure}$ must reach values higher than the ultimate glass transition temperature, $T_{g\infty }$, in order to overcome the effect of vitrification and ensure completion of the curing process.

The transformations taking place during curing processes are commonly represented in conversion-temperature-transformation (CTT) diagrams [10] or time-temperature-transformation (TTT) diagrams [15]. These diagrams serve not only to identify the different structural states through which the system can pass, but also to design temperature programs in accordance with the curing kinetics and process requirements. For practical purposes, the curing kinetics are usually analyzed using phenomenological methods. The effect of vitrification can be taken into consideration if higher accuracy is required. The evolution of $T_{g}$ during the reaction can also be modelled using suitable approximations, rather than a detailed structure-based analysis.

In consequence, it is essential to have a set of basic tools that can be used to characterize a curing process and have a comprehensive picture of it, which can be exploited for the efficient design of curing processes and to tailor the final structure/properties. In the following subsections, some common methods will be outlined.

### 2.1. Network Build-Up Analysis

**Figure 2:**
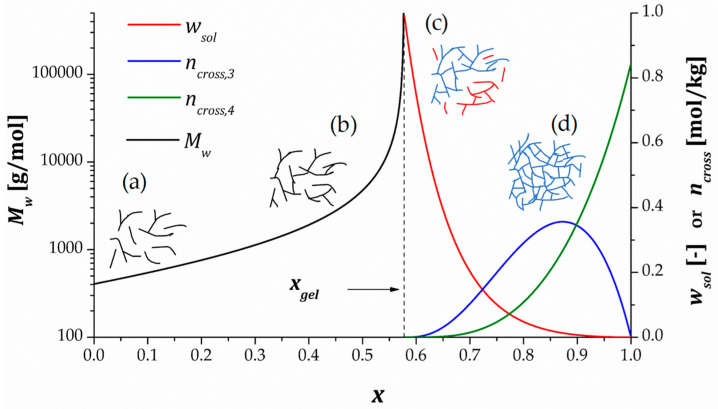
Evolution of network build-up statistical averages with degree of conversion x for an ideal $A_{2}-B_{4}$ stepwise copolymerization.

As mentioned, the stepwise mechanism of click polymerization is highly beneficial in thermoset curing in terms of structure and network build-up. Unlike chain-wise polymerizations in which monomer units are added sequentially to the growing chain, stepwise processes proceed ideally by successive and random coupling of fragments with gradually increasing size by reactions between complementary A and B functional groups. Ideal stepwise behavior implies equal reactivity of all groups and that no intramolecular cyclization takes place. Figure 2 illustrates the evolution of the molecular and network architectures with respect to the extent of the reaction or degree of conversion x for the curing process of a stoichiometric mixture of a bifunctional A monomer, $A_{2}$, and a tetrafunctional B monomer, $B_{4}$. Since the reaction system is stoichiometric, the degree of conversion x is the same for groups A and B. Otherwise, it can be specified whether x refers to group A or B, or else it can be ascribed to the conversion of the limiting reactant, which will control the extent of the reaction. The specific data shown in the graph would obviously change for a different curing system, but the overall features of the network build-up process are very similar for any crosslinking system.

As seen in Figure 2, at the beginning of the process, (a) low molecular weight species (i.e., monomers or oligomers) predominate in the system. The progress in the reaction leads to the formation of (b) species with increasing size and degrees of branching. This process takes place with increasing mass-average molecular weight $M_{w}$ and a broadening of the polymer distribution. At some point, this increasing molecular growth and branching will end up in the formation of a giant macromolecule with infinite $M_{w}$ percolating the bulk of the material, leading to the formation of (c) an incipient network structure. This is known as gelation, and usually takes place at a fixed degree of conversion $x_{gel}$ irrespective of reaction conditions. Given that $M_{w}$ diverges to infinity at $x_{gel}$, the viscosity of the system becomes infinite, and therefore, the system ceases to flow. In addition, it starts to become insoluble so that the soluble fraction $w_{sol}$ becomes lower than 1. The formation of the gel implies the presence of crosslinking points, interconnected by network strands, so that a crosslinking density $n_{cross}$ may be defined. Once the gel point is passed, the reaction continues with decreasing soluble fraction $w_{sol}$ and increasing crosslinking density, $n_{cross}$. It should be noted that, for an $A_{2}-B_{4}$ copolymerization, trifunctional crosslinks ($n_{cross,3}$ in Figure 2) are mainly formed right after gelation, but eventually, these turn into tetrafunctional crosslinks ($n_{cross,4}$ in Figure 2) until all reactive groups have been exhausted, leading to (d) the ultimate, fully crosslinked and insoluble network structure.

The evolution of the molecular and network structure during a stepwise polymerization process can be easily analyzed making use of well-established mean-field methods, based on combinatorial [16] or recursive approaches [10,13,17,18,19] which are all equivalent. We will just outline some key aspects of the recursive methodology based on structural fragments. A comprehensive and detailed explanation of this method can be found in the literature [10,18].

**Scheme 2:**
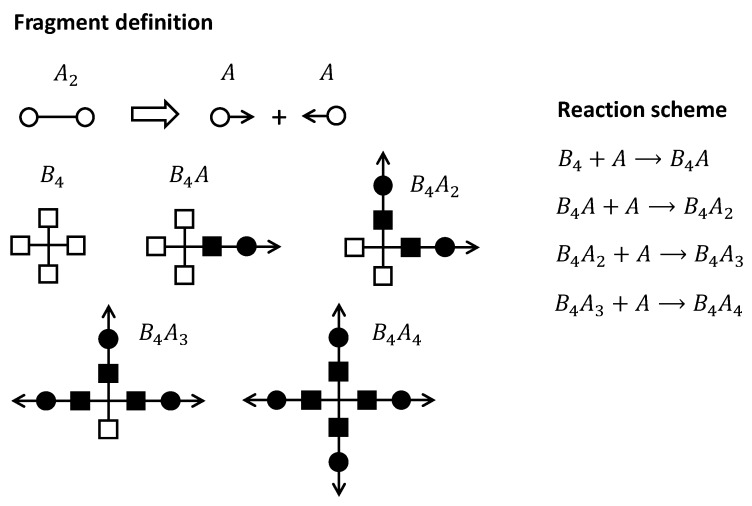
Definition of structural fragments for the network build-up analysis of an $A_{2}-B_{4}$ ideal stepwise copolymerization.

The starting point of the recursive method is to define a set of structural fragments which are representative of the different states of reaction that can be found for the different monomers. For instance, for an $A_{2}$ + $B_{4}$ reactive system, the set of structural fragments shown in Scheme 2 can be defined.

Reactive groups A have been identified with an empty circle, and reactive groups B with an empty square. The $A_{2}$ monomer is split into two A fragments that have an arrow, which indicates that they are, in reality, connected. Now, it can be seen that when a $B_{4}$ monomer reacts with an A fragment, a new structural fragment, $B_{4}A$, can be defined, which has a reacted B group (filled square) attached to a reacted A group (filled circle); this fragment has an arrow coming from the reacted A fragment. If the $B_{4}A$ reacts with another A fragment, we obtain another structural fragment, $B_{4}A_{2}$, issuing 2 arrows from the reacted A fragments, and so on, in order to obtain fragments $B_{4}A_{3}$ and $B_{4}A_{4}$. In the case of ideal stepwise polymerization, the concentration of the different structural fragments can be easily obtained using the binomial distribution, taking into account the global conversion of groups A and B and the number of reacted/unreacted sites of each fragment.

**Scheme 3:**
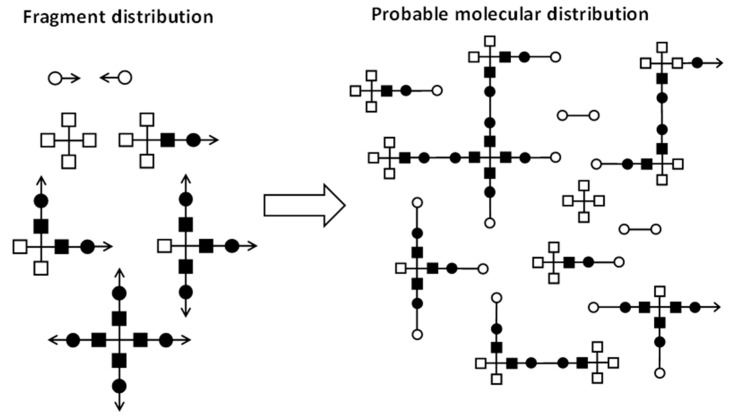
Random combination of structural fragments into probable molecular distribution for the network build-up analysis of an $A_{2}-B_{4}$ ideal stepwise copolymerization.

Now, the key is to understand that all the reactive fragments carrying at least an arrow can be randomly recombined with any other fragment carrying another arrow. In this way, a most probable distribution of polymeric species within the reactive system can be created, as indicated in Scheme 3. However, it is not the purpose of these methods to determine the exact polymer distribution, but to calculate key structural parameters such as $M_{w}$ before gelation, or $w_{sol}$ and $n_{cross}$ after gelation, and, of course, the gel point conversion, $x_{gel}$. Before gelation, the random recombination of fragments leads to a distribution of species with finite size. That means that the *expected mass* pending from an arrow bond is finite. In other words, the probability that the path starting from an arrow is finite, or the *extinction probability*, is equal to 1. The *expected mass* increases as the reaction advances, and diverges to infinity when gelation takes place. Pregel parameters such as $M_{w}$ are determined from the *expected mass*. Once gelation takes place, the presence of a gel fraction with infinite molecular weight causes the *extinction probability* to have a value lower than 1. Structural parameters after gelation such as $w_{sol}$ and $n_{cross}$ are derived from the *extinction probability*. The value of the *extinction probability* decreases as the reaction advances, and in consequence, $n_{cross}$ increases and $w_{\mathrm{sol}}$ decreases. Ideally, it could reach a value of 0 at the end, indicating that complete crosslinking has been reached and there is no soluble fraction, but under some circumstances (i.e., off-stoichiometric systems, nonideal stepwise reactions or the presence of impurities with lower functionality), it leads to nonzero values at the end, resulting in a soluble fraction, incomplete crosslinking and other network defects.

These two concepts, *expected mass* and *extinction probability*, are determined before and after gelation, respectively, using a recursive procedure that takes into account the probability of picking any fragment issuing a connecting arrow, and the remaining connections with other fragments, if any [10,18]. It should be noted that although other mathematical descriptions can be used in order to analyze the process, all mean-field methods produce equivalent results. Depending on the complexity of the system, alternative definitions of fragments with a more specific labelling of their connections are also possible and necessary, taking into account the connection possibilities and the internal structure of the crosslinking agent [10,18,20,21].

When it comes to the design of a crosslinking system, the main concern is whether the process will produce a crosslinked network or not, and the conversion of reactive groups A and B at gelation. To start with, let us imagine that we are reacting $A_{f}$ and $B_{g}$ molecules bearing functional groups A and B with functionalities f and g, respectively. In addition, we might not necessarily be under stoichiometric reaction conditions. Therefore, we should determine the conversion of groups A and B, which can be defined as:

$$x_{A}=1-\frac{n_{A}\cdot f}{n_{A,0}\cdot f}=1-\frac{n_{A}}{n_{A,0}}\;x_{B}=1-\frac{n_{B}\cdot g}{n_{B,0}\cdot g}=1-\frac{n_{B}}{n_{B,0}}$$

where $n_{A,0}$ and $n_{B,0}$ are the initial concentration or number of monomers A and B, respectively, and $n_{A}$ and $n_{B}$ are the concentrations of the A and B momoners at a given time. Given that the number of reacted A groups must equal the number of reacted B groups, $x_{A}$ and $x_{B}$ are related by the feed ratio of A and B functionalities $r_{B/A}$ in the following way:

$$r_{B/A}=\frac{n_{B,0}\cdot g}{n_{A,0}\cdot f}\;x_{B}=\frac{x_{A}}{r_{B/A}}$$

For an ideal system, gelation for a stepwise condensation between $A_{f}$ and $B_{g}$ molecules, the following condition is fulfilled before gelation takes place [10,18]:

$$x_{A}\cdot x_{B}<\frac{1}{\left(f-1\right)\cdot \left(g-1\right)}$$

and the condition for gelation is given by:

$${\left(x_{A}\cdot x_{B}\right)}_{gel}={\left(x_{A}\cdot \frac{x_{A}}{r_{B/A}}\right)}_{gel}=\frac{1}{\left(f-1\right)\cdot \left(g-1\right)}\Rightarrow \;x_{A,gel}=\sqrt{\frac{r_{B/A}}{\left(f-1\right)\cdot \left(g-1\right)}}$$

For the example discussed in the figure, a stoichiometric mixture ($r_{B/A}=1$) of $A_{2}$ and $B_{4}$ monomers, with f=2 and g=4, leads to $x_{A,gel}=x_{B,gel}=0.57735$. Because the system is stoichiometric, $x_{gel}=x_{A,gel}=x_{B,gel}$; the system becomes fully insoluble and the crosslinking density reaches its maximum at the end of the process.

However, given that stepwise reactions are self-limiting, one could vary the feed ratio between the monomers with the purpose of obtaining a partially reacted system with a controlled excess of groups A or B. Depending on the initial feed ratio of the monomers, $r_{B/A}$, the resulting material at the end of the process would be partially crosslinked, or else it would not be gelled and would retain solubility and flowability. For these systems, it is important to define the critical ratio at gelation, which is the limiting feed ratio that leads to gelation when conversion of groups A or B is complete. When the A monomer is in excess, B groups will react completely at the end of the process:

$$r_{B/A}<1\Rightarrow x_{B}=1\Rightarrow x_{A}=r_{B/A}$$

$${\left(x_{A}\cdot x_{B}\right)}_{gel}={\left(x_{A}\cdot \frac{x_{A}}{r_{B/A}}\right)}_{gel}=r_{B/A,crit}=\frac{1}{\left(f-1\right)\cdot \left(g-1\right)}$$

This means that if $r_{B/A}<r_{B/A,crit}$, the system will not gel when all B groups have reacted. For an $A_{2}$ and $B_{4}$ stepwise polymerization, with f=2 and g=4, $r_{B/A,crit}$ has a value of 1/3. Conversely, when the B monomer is in excess:

$$r_{B/A}>1\Rightarrow x_{B}=1/r_{B/A}\Rightarrow x_{A}=1$$

$${\left(x_{A}\cdot x_{B}\right)}_{gel}={\left(x_{A}\cdot \frac{x_{A}}{r_{B/A}}\right)}_{gel}=\frac{1}{r_{B/A,crit}{}^{\prime }}=\frac{1}{\left(f-1\right)\cdot \left(g-1\right)}$$

$$r_{B/A,crit}{}^{\prime }=\left(f-1\right)\cdot \left(g-1\right)$$

In consequence, when $r_{B/A}>r_{B/A,crit}{}^{\prime }$, the system will not gel when all A groups have reacted. In the present case, $r_{B/A,crit}{}^{\prime }=3$.

**Figure 3:**
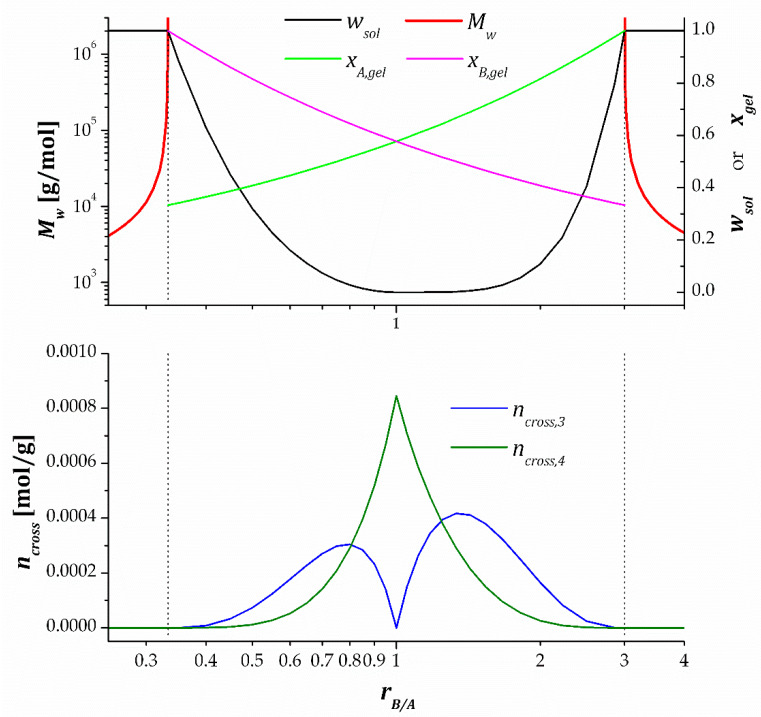
Evolution of feed ratio of functionalities A and B, $r_{B/A}$, on build-up statistical averages for an ideal $A_{2}-B_{4}$ stepwise copolymerization. The dashed vertical lines on both graphs represent the critical ratios $r_{B/A,crit}=1/3$ and $r_{B/A,crit}{}^{\prime }=3$.

Figure 3 illustrates the effect of varying $r_{B/A}$ on the relevant statistical averages determined for an ideal of $A_{2}$ and $B_{4}$ stepwise polymerization. The upper graph shows the evolution of $M_{w}$, $w_{sol}$ at the end of the reaction, and degrees of conversion at gelation $x_{A,gel}$ and $x_{B,gel}$, while the lower graph gives insight into the network crosslinking density at the end of the process. Two vertical dashed lines have been plotted on each graph, corresponding to the critical ratios $r_{B/A,crit}=1/3$ and $r_{B/A,crit}{}^{\prime }=3$.

It can be clearly observed that at $r_{B/A}<r_{B/A,crit}$, the system is not gelled and fully soluble $w_{sol}=1$, with growing average molecular weight and diverging to infinity as $r_{B/A}$ approaches $r_{B/A,crit}$. At $r_{B/A}=r_{B/A,crit}=1/3$, the system gels at the very end of the process, with $x_{B,gel}=1$ and $x_{A,gel}=1/3$. The soluble fraction decreases with increasing $r_{B/A}$ until it reaches a minimum value, i.e., $w_{sol}=0$ at $r_{B/A}=1$, under stoichiometric conditions. At this point, $x_{A,gel}=x_{B,gel}=x_{gel}=0.57735$. Increasing $r_{B/A}$ leads to a growing soluble fraction until $r_{B/A}=3$, when gelation takes place at the very end of the process, with $x_{A,gel}=1$ and $x_{B,gel}=1/3$, and $w_{sol}=1$. A further increase of $r_{B/A}$ leads to ungelled polymers with decreasing $M_{w}$. With regards to the crosslinking density, the lower graph shows that there are no crosslinks when $r_{B/A}<r_{B/A,crit}=1/3$ or $r_{B/A}>r_{B/A,crit}{}^{\prime }=3$. Between $r_{B/A,crit}$ and $r_{B/A,crit}{}^{\prime }$, trifunctional and tetrafunctional crosslinks are found in the system, with only tetrafunctional crosslinks and maximum crosslinking density at $r_{B/A}=1$, as outlined before. 

Figure 3 also illustrates the effect of unbalanced stoichiometry caused by uncertainty in the monomer structure and functionality. The graphs are clearly asymmetrical with respect to $r_{B/A}$. It can be appreciated that it is better to formulate materials with a slight excess of the tetrafunctional monomer $B_{4}$, $r_{B/A}>1$, because the soluble fraction $w_{sol}$ is nearly 0 and the crosslinking density is near its maximum. In contrast, if a small excess of the difunctional monomer $A_{2}$, $r_{B/A}<1$, the crosslinking density drops more dramatically, and the soluble fraction increases significantly.

It should be noted that the preceding discussion can be extended to other systems with different functionalities of A and B monomers, or else to a more general case, when mixtures of A or B monomers with different functionalities are combined. The expressions for gel point conversion and critical ratios are identical to those shown before, but using the average functionalities $f_{A,e}$ and $g_{B,e}$ instead [10,18]. A full detailed analysis of the general case for the ideal stepwise behavior can be found in the literature [18]. If monomers with mixed functionality AB were used (e.g., as in the synthesis of hyperbranched structures using $AB_{2}$ monomers [22]), it would still be possible to apply the methodology, but the definition of structural fragments would be more complex than the one shown in the figure, and should take into consideration the explicit connections between the different fragments using suitable labelling [23].

In consequence, there is a great potential in material design and tailoring, using simple design parameters such as the monomer feed ratio and functionality. Any material with a controlled excess of monomers may be desirable, especially when a second polymerization takes place or some surface functionality is required, as in dual-curing systems [24,25,26,27], or for the synthesis of prepolymers that will later be mixed with other curing agents. In any case, materials with controlled and predictable network architecture can be designed [19].

However, deviations from this predicted ideal stepwise behavior occur frequently as a consequence of a number of non-idealities [10], mainly:

*(a)*  *Unequal reactivity of the groups in the monomer*, i.e., by substitution effects. Negative substitution effects are common in reactive systems based on epoxy reactions with aromatic amines [28], or aza-Michael addition with aliphatic amines [29,30], in which the reactivity of a secondary amine hydrogen decreases significantly after the reaction of a primary amine. The effect is to delay branching and gelation. This effect has been studied in detail in the literature using different methodologies [10,31,32]. The effect of negative substitution effects in terms of gel point conversion may be difficult to determine because differences may fall within the experimental error range, but it may be much easier to determine it in terms of the critical ratio at gelation [10]. In general, it can be stated that $r_{B/A,crit}<1$ would increase in the presence of an excess A monomer with a negative subsitution effect. Conversely, $r_{B/A,crit}{}^{\prime }>1$ would decrease in the presence of an excess of a B monomer with a negative substitution effect.
*(b)*  *Intramolecular cyclization reactions* take place when there is a nonnegligible probability that a reactive A group meets a reactive B group from the same molecule. In consequence, the reaction takes place without an increase in molecular weight or branching, thereby delaying gelation. The presence of cyclic structures may lead to the presence of network defects such as dangling chains and a soluble fraction at the end of the reaction, and to an effective reduction in crosslinking density and equilibrium modulus [31,33,34,35], and this is further aggravated in diluted systems because of the reduced probability of intermolecular reactions. Some reactive systems have an intrinsic tendency to form intramolecular cycles due to the particular flexibility of some of the reactants [31,32,33,36]. Some approximations to the determination of the extent of cyclization based on the spanning-tree concept have been described in the literature [30,35] and references therein. Parameters such as $x_{gel}$ or $r_{B/A,crit}{}^{\prime }$ have a fairly linear dependence with the inverse of the concentration of reactive groups 1/c [31,33], thereby making it possible to determine the ring-free (i.e., no cyclization) parameters by extrapolating c→∞.
*(c)*  *The formation of preferred nonrandom structures*, i.e., driven by thermodynamics, is also described in the literature, such as silanol condensation or cyclotrimerization reactions [10,37]. A combination of structural fragments becomes nonrandom, and therefore, larger preferred structures are formed. This may be taken into consideration using a suitable kinetic-structural model, so that these larger structures can be incorporated into the recursive model.

The values of $x_{gel}$ coming from the ideal stepwise approach can be easily verified with rheological [36,38] or thermomechanical analysis [39,40] in order to determine the time for gelation or solubility measurements [41], in combination with an experimental measurement of conversion using techniques such as differential scanning calorimetry (DSC) or Fourier-transform infra-red spectroscopy (FTIR). Under off-stoichiometric conditions, the ratio of functionalities $r_{B/A}$ and the existence of a limiting reactant should be taken into consideration for the correct determination of individual degrees of conversion of groups A and B. In this way, nonidealities, if present, can be easily identified [21,36]. Some nonidealities such as substitution effects can be incorporated into the network build-up model and verified experimentally [41]. Although different reaction conditions can be used for the measurement of $x_{gel}$, it has been experimentally verified that the gelation in stepwise systems is an iso-conversional phenomenon [10,39] and therefore predictions of gel times can be reasonably made if a sound kinetic model is used. Soluble fraction ($w_{sol}$) measurements [31] or average molecular weight determinations [41] can also be performed in order to validate the model predictions. 

To conclude, mean-field recursive methods can be used to make preliminary estimates of the network build-up process and relevant parameters such as $x_{gel}$, critical ratios $r_{B/A,crit}$ or $w_{sol}$, but experimental validation is compulsory in order to determine departures from the ideal model. The question of whether these departures may be incorporated into a more complex model or they are just used and taken into consideration from a practical point of view is not relevant, as long as the information is used consistently.

### 2.2. Glass Transition-Conversion Relationships

The evolution of $T_{g}$ with the extent of reaction or degree of conversion x, hereafter $T_{g}\left(x\right)$, is crucial from a processing point of view. If the $T_{g}$ of the reactive system increases sufficiently to reach the curing temperature $T_{cure}$, this reduction in mobility will slow the curing significantly, eventually halting it, leading to an incomplete cure and inferior material properties. In this case, the degree of conversion x is generally defined from a phenomenological point of view (i.e., fractional reaction heat evolved in a curing process), which is assumed to be proportional to the extent of the reaction [12]. If the system is stoichiometric, x is equivalent to the conversion of groups A and B. If not, it is equivalent to the conversion of the limiting reactant, which will dictate the extent of the reaction. Experimental determination of $T_{g}\left(x\right)$ relationships is usually carried out from partial curing measurements, using differential scanning calorimetry (DSC) [12,41]. Although curing processes can be complex, it is generally acknowledged that there is a unique relationship between the glass transition temperature and the degree of conversion of a given curing system $T_{g}\left(x\right)$, mainly independent of the processing conditions [10,12,14,40,42]. Such a relationship generally has a curved shape with steeper slope towards the end of the curing process, as commonly reported in the literature [14,42].

Given that the evolution of $T_{g}\left(x\right)$ is connected with the structural changes that take place during curing, several structure-based approaches have been used to model the $T_{g}\left(x\right)$ relationship [14]. However, interesting as it is, such approaches generally require an exhaustive determination of experimental $T_{g}\left(x\right)$ data, a precise knowledge of the network build-up process, and additional adjustment of semi-empirical parameters to the experimental data. In cases where this is impractical, any expression fitting the experimental $T_{g}\left(x\right)$ data might be used instead.

In consequence, it is of interest to derive some other expressions that can be used to model or predict the $T_{g}\left(x\right)$ relationships making use of a reduced set of experimental data. Two useful approaches have been reported in the literature [42,43], based on the thermodynamic theory of Couchman for polymer mixtures [44]. These methods assume that the $T_{g}$ of a partially reacted system can be determined additively from a compatible blend of the unreacted monomer mixture with $T_{g0}$, and the fully cured polymer with $T_{g\infty }$.

The expression derived by Pascault and Williams [43] has the following form:

$$\frac{T_{g}-T_{g0}}{T_{g\infty }-T_{g0}}=\frac{\lambda \cdot x}{1-\left(1-\lambda \right)\cdot x}$$

where $T_{g\infty }$ is the glass transition temperature of the fully cured polymer network, $\lambda =\Delta C_{p\infty }/\Delta C_{p0}$. $\Delta C_{p\infty }$ is the heat capacity step during the glass transition of the fully cured polymer network, and $\Delta C_{p0}$ is the heat capacity step during the glass transition of the unreacted mixture of monomers. This is a highly useful expression which enables the determination of the full $T_{g}\left(x\right)$ relationship, making use of only two experimental measurements before and after the curing process.

In contrast, the expression by Venditti and Gillham [42] has a different form:

$$lnT_{g}=\frac{\left(1-x\right)\cdot lnT_{g0}+\lambda \cdot x\cdot lnT_{g\infty }}{\left(1-x\right)+\lambda \cdot x}$$

where the parameter $\lambda =\Delta C_{p\infty }/\Delta C_{p0}$ has exactly the same meaning as in the previous expression. The difference of this expression is in the consideration of whether $\Delta C_{p}$ is constant with temperature T [43] or else $\Delta C_{p}\cdot T=\Delta C_{p.T=T_{g}}\cdot T_{g}$ [42]. It has been reported that both expressions can be used to fit experimental $T_{g}\left(x\right)$ relationships of different thermosetting systems using λ as an adjustable parameter. However, the expression by Venditti and Gillham has better predictive capabilities, since the adjusted λ is much closer to the experimentally measured $\lambda =\Delta C_{p\infty }/\Delta C_{p0}$ [42] and is therefore recommended in order to estimate the $T_{g}\left(x\right)$ relationship using the smallest possible set of experimental measurements.

### 2.3. Temperature-Transformation Diagrams

The transformations taking place during a curing process may be conveniently represented in conversion-time-temperature (CTT) and time-temperature-transformation (TTT) diagrams, which are used to identify the states in the processing of a thermoset [10,15]. In addition, the TTT diagram includes kinetic information that can be used to plan curing processes under isothermal conditions or in multiple isothermal steps [45].

**Figure 4:**
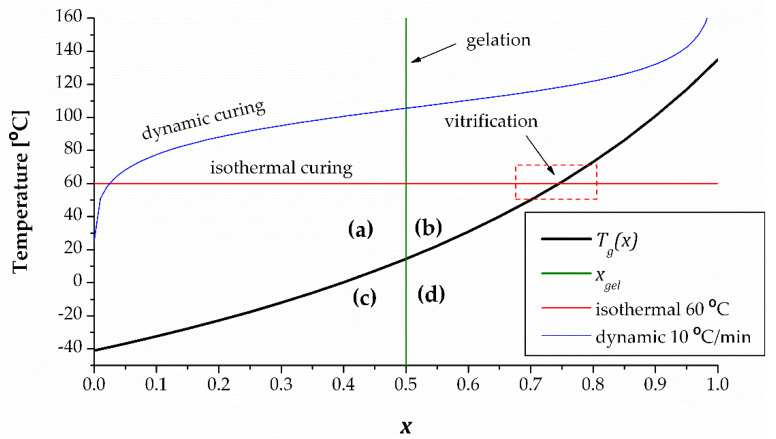
Conversion-temperature-transformation (CTT) diagram for a DGEBA-diethylenetriamine system, produced from the data reported in the literature [46].

Figure 4 shows an example of CTT diagram determined from the data reported for a stoichiometric DGEBA-diethylenetriamine formulation [46], which would be equivalent to an $A_{2}-B_{5}$ with $r_{B/A}=1$. 

In this diagram, the degree of conversion x has been determined using DSC. Given that the system is stoichiometric, x represents the conversion of either epoxy or amine groups. If the system were nonstoichiometric, it would be equivalent to the conversion of the limiting reactant controlling the extent of reaction, as discussed before. The vitrification line or $T_{g}\left(x\right)$ relationship determined using the Pascault and Williams expression [43] is represented, and the gelation line corresponding to $x_{gel}=0.5$ has been determined assuming ideal stepwise behavior. The $T_{g}\left(x\right)$ has an upwards trend with a steeper slope towards the end, as expected [14], and the gelation line is vertical, which comes from the assumption that gelation is independent from the curing temperature program. The intersection between the vitrification and gelation lines divides the CTT diagram into 4 regions: (a) at $x<x_{gel}$ and $T>T_{g}\left(x\right)$ the system is ungelled and a flowing liquid (b) at $x>x_{gel}$ and $T>T_{g}\left(x\right)$ the system is a gelled rubber, i.e., soft but not flowing, (c) at $x<x_{gel}$ and $T<T_{g}\left(x\right)$ the system is an ungelled glass, i.e., still liquid but solid-like because the flow is arrested by vitrification, and (d) at $x>x_{gel}$ and $T<T_{g}\left(x\right)$ the system is a gelled glass, i.e., hard solid, nonflowing material. In addition, it is also useful to plot isothermal and nonisothermal curing lines in the diagram. In the figure, it can be observed that an isothermal curing process is represented as a horizontal line. It can be seen that because of $T<T_{g\infty }$, the curing line intersects the vitrification line. This has a significant impact in terms of the kinetics and completion of the cure. As the curing approaches vitrification, the kinetics will become progressively diffusion-controlled rather than chemically controlled, and the process will slow down significantly. In practical terms, the system may reach a somewhat higher extent of cure than that predicted by the $T_{g}\left(x\right)$ relationship [46], but the curing will halt and will remain incomplete. It would be necessary to raise the temperature up to values higher than $T_{g\infty }$ in order to ensure completion of the cure. In addition, because the curing process will already have crossed the gelation line, the system would have lost its flowing ability. If one wanted to partially cure a material but retain its flowing ability, it would be necessary that $x<x_{gel}$. This diagram can also be used to determine safe storage conditions in order to prevent gelation. The intersection between the vitrification and gelation lines takes place at a specific temperature,$\;T_{g,gel}$. This would be the limiting storage temperature so that the system will not gel upon storage. In the diagram, a dynamic curing line is also drawn. Because of an ever-increasing curing temperature which would always be higher than $T_{g}\left(x\right)$, the curing process would inevitably reach completion. The curing curve would approach $T_{g}\left(x\right)$ only at very low heating rates, and would thus be affected by vitrification [46,47]. Nevertheless, the ongoing increase in temperature would ensure the completion of the cure anyway.

**Figure 5:**
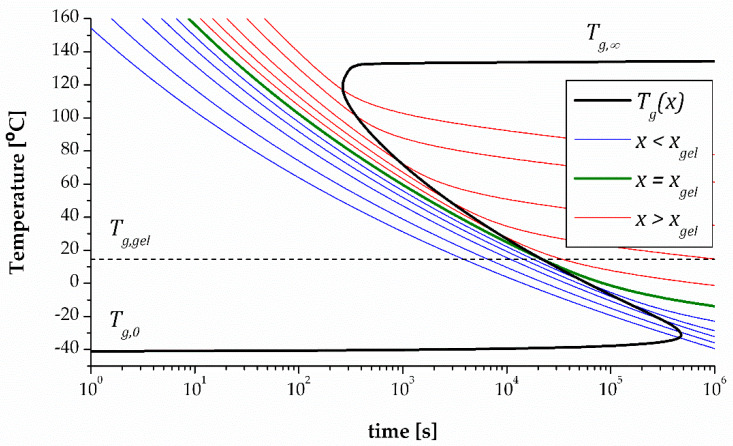
Time-temperature-transformation (TTT) diagram for a DGEBA-diethylenetriamine system, produced from the data reported in the literature [46].

An example of TTT diagram produced from the same data [46] is shown in the Figure 5.

Logarithmic time scales are generally used to account for the temperature dependence of the curing rate. The degree of conversion x is defined in the same way as before. Iso-conversional lines calculated under isothermal conditions are plotted, before gelation $x<x_{gel}$ (in blue), at gelation $x=x_{gel}$ (in green) and after gelation $x>x_{gel}$ (in red). Iso-conversional lines are parallel, and they generally spread out when they cross the vitrification line due to the effect of vitrification on kinetics (if this is taken into consideration in the analysis). The vitrification line, which unites all the points at which $T_{cure}=T_{g}\left(x\right)$, is not iso-conversional, and has a characteristic S-shape because of the significant increase in curing rate with increasing temperature. It spans from the initial glass transition temperature $T_{g0}$ to the ultimate glass transition temperature $T_{g\infty }$. Several methods are available to determine the curing kinetics and the vitrification line for a TTT diagram [41,46,48]. As in the CTT diagram, several states can be identified depending on whether the conversion is lower or greater than $x_{gel}$, or the temperature is higher or lower than $T_{g}\left(x\right)$. The introduction of the time variable by means of the kinetics data makes it possible, in addition, to define curing schedules according to specific process requirements, in single or multiple curing stages [45]. More exotic shapes of TTT diagram can be obtained in complex systems such as dual-curing systems [49].

## 3. Preparation of Thermosets by Nucleophilic Attack to Epoxy Resins

Cross-linked epoxy thermosets exhibit outstanding properties and constitute the best alternative in structural applications, such as adhesives, coatings and composites. This is mainly because of the flexibility in the structure and/or molecular weight of the starting epoxy compounds, and in the selection of the type and structure of the curing agents. These materials present excellent adhesion properties, high thermal and environmental stability, low contraction on curing and superior mechanical characteristics [50,51].

Epoxy resins can be cured with a broad variety of curing agents by a typical polycondensation process (amines, acids, phenols, dicyandiamide, etc.) or by the homopolymerization of epoxides using basic or acid initiators, following a ring-opening polymerization mechanism. Even though the reaction of diepoxy compounds with amines and mercaptans was reported in the patent literature in the 1930s, their commercialization only started in 1947. In fact, epoxy curing systems are examples of what are known today as click reactions in the preparation of thermosetting materials.

**Scheme 4:**
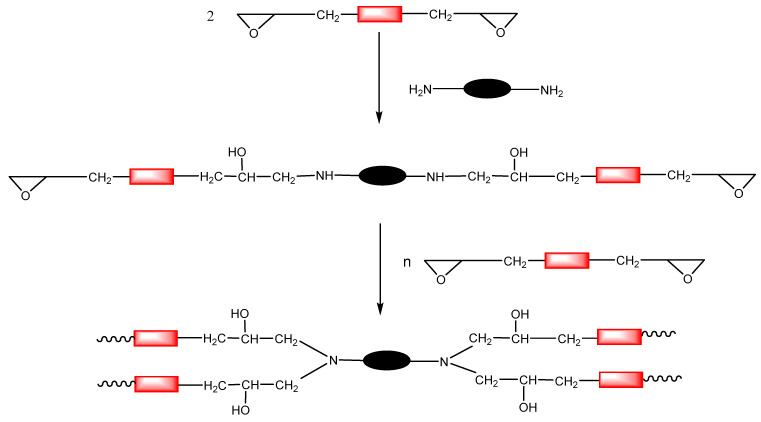
Curing process by epoxy-amine click reaction.

Primary and secondary amines can react with epoxides by nucleophilic attack to the less hindered carbon of the epoxy ring, following a click pattern (see Scheme 1) [52]. The formation of a network from a diepoxy compound requires an amine with a functionality of at least 3; a functionality of 4 is usually preferred. In this case, a primary diamine is used as a hardener to perform the crosslinking process. Primary amines are more reactive than secondary amines, and for this reason, the curing proceeds by the formation of linear oligomers that react to form the network in the last stages of the reaction process. Scheme 4 depicts the formation of the network by the epoxy-amine click reaction.

Aliphatic amines have higher nucleophilicity than aromatics; therefore, the former cure at lower temperatures [53,54]. Resins that have been cured using aliphatic amines are strong and present excellent bonding properties. They are resistant to alkalis and some inorganic acids, and show good resistance to water and solvents, but are not so resistant to many organic solvents. To increase the curing rate, sometimes a combination of aliphatic and aromatic amines is used [55]. Aromatic diamines are less reactive, and the curing usually stops at the formation of a linear polymer due to the large difference in the reaction of primary and secondary amines [56]. However, upon increasing the temperature to 150–170 °C, curing continues to completion. Aromatic amines provide excellent heat resistance and good mechanical and electrical properties. In addition, the resulting thermosets have excellent chemical resistance, especially to alkalis, and they are highly resistant to solvents.

**Scheme 5:**
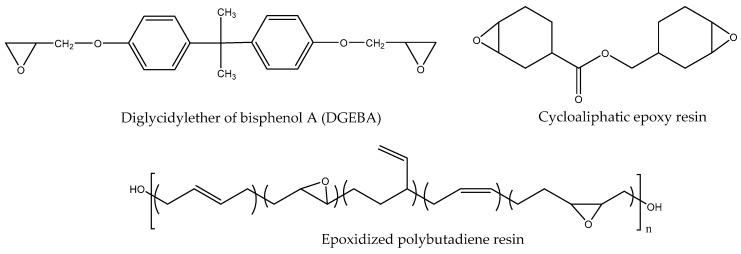
Structures of different type of epoxy resins.

The rate of curing of epoxide with amines depends not only on the amine structure, but also on the epoxide characteristics. The most commonly used diglycidylether type resins easily cure at room temperature with aliphatic amines, but hindered epoxies such as cycloaliphatic epoxide and epoxidized rubbers hardly cure in these conditions (see Scheme 5).

Epoxy resins based on renewable starting materials have also been cured by a variety of amines [57,58]. Among these renewable materials are oils [59], eugenol [60], vanillin [61] and magnolol [62].

Epoxy-amine reaction was also used in off-stoichiometric mixtures, as a first curing stage in dual curing processes. Therefore, these systems are only click in the first stage, as epoxy homopolymerization reactions (which occur in the second stage) do not fit the click criteria. As the secondary epoxy homopolymer network imparts high crosslink density to the pristine epoxy-amine network, this dual-curing strategy is useful to obtain materials with superior mechanical properties. Secondary epoxy homopolymerization, with well-defined curing kinetics, is usually carried out using anionic initiators such as imidazole derivatives [63,64]. Since the epoxy-amine reaction has a self-limiting character, the intermediate materials can be stored safely for more than 50 days. Such dual-curable epoxy formulations are reviewed more in detail in Section 9.

**Scheme 6:**
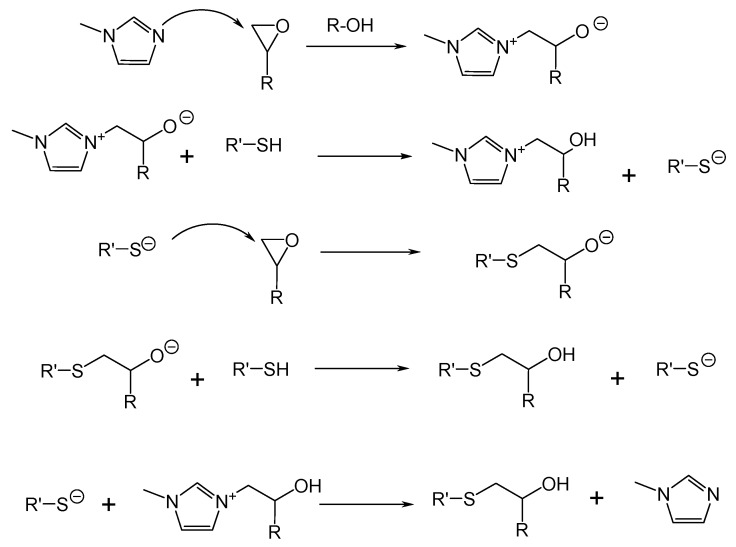
Thiol-epoxy reaction catalyzed by 1-methylimidazole as tertiary amine.

Although not as common as amines, thiols are also employed as epoxy curing agents. Thiol-epoxy reactions require the presence of an amine as the catalyst. The amine can act directly as a base, extracting the thiol proton and enhancing the nucleophilicity by forming thiolate active groups, or as a nucleophile, attacking to the epoxide and finally generating thiolate functions, as depicted in Scheme 6. The use of an amine catalyst usually leads to an extremely fast reaction and to a short pot-life. In order to prolong pot-life and facilitate the application of the formulation, latent catalysts can be used, which, when triggered, generate a base. The activation can be performed thermally or photochemically [65,66].

The higher nucleophilicity of thiolates in comparison to amines facilitates their reaction with cycloaliphatic epoxides [67] and internally epoxidized linear chains [68]. Thiolates derived from thiophenols react much faster due to their higher nucleophilicity [68].

The fast thiol-epoxy reaction in the presence of tertiary amines, together with the high fluidity of the thiol monomers, has been utilized to obtain self-healing materials by the addition of two types of microcapsules. One microcapsule contains a thiol/amine mixture and the other the epoxy resins. When a crack occurs in the polymer film, the microcapsules break open and release their components which react and heal the crack instantaneously [69]. This self-healing strategy is reviewed in Section 10.

Thiol-epoxy curing based on renewable monomers like eugenol [70,71], phloroglucinol [72] or gallic acid [73] has also been recently reported.

The thiol-epoxy reaction has been used in the preparation of polymer matrix in nanoclay [74] and boron nitride [75,76] composites. In the latter, the thermal conductivities achieved were higher than in amine-epoxy composites.

Similarly, off-stoichiometric thiol-epoxy formulations can also be dual-cured using adequate initiators. Our research group has prepared such materials, described their curing kinetics and fully characterized their shape-memory capabilities [25,36,77]. We showed that depending on the excess of epoxy in the initial formulation, intermediate materials can either be fluids or gels, similar to the off-stoichiometric epoxy-amines mentioned before. However, their shape-memory capabilities are superior to those of epoxy-amine materials.

## 4. Preparation of Thermosets by Michael Addition

Michael-type additions have long been utilized in organic synthesis and more recently in materials science, but relatively few implementations have been demonstrated in the preparation of cross-linked polymer networks. In the last twenty years, the Michael reaction has been more frequently encountered in this field, due to its click nature that yields uniform and homogeneous thermosets in mild and environmentally friendly conditions.

Michael additions are 1,4-addition reactions of α, β–unsaturated carbonyl compounds and α, β–unsaturated nitriles with resonance-stabilized carbon nucleophiles, such as enolate ions and enamines. In this thermodynamically favored reaction, during the strong nucleophilic attack on the β-carbon of an α, β-unsaturated carbonyl, a negatively charged intermediate is formed, which subsequently yields the Michael adduct by protonating the catalyst. The α, β–unsaturated compound undergoing Michael addition is called Michael acceptor, the nucleophile is called Michael donor and the product is called Michael adduct. The most typical Michael donors are thiols (thiol-Michael), primary and secondary amines (aza-Michael), alcohols (oxa-Michael) and acetoacetates (carbon-Michael) that easily generate C-S, C-N, C-O and C-C bonds, respectively.

Common Michael acceptors, are electron-deficient *enes* such as maleimides, vinyl sulfones, acrylates/acrylamides and methacrylates, in decreasing order of reactivity, among others [29,78]. Michael additions show selectivity towards the strong electron-withdrawing groups, making it possible to perform sequential reactions in formulations containing *enes* with different acceptor abilities [79,80,81,82,83]. Another relevant feature of the Michael reaction, due to its click nature, is its self-limiting reactivity which makes it possible to work with Michael donors in excess, i.e., off-stoichiometric amounts. In such formulations, after the first stage of Michael addition, the excess of Michael donors can be polymerized, giving the material its final properties. This strategy is extremely useful when the second stage can be activated independently by irradiation [24,84].

**Scheme 7:**
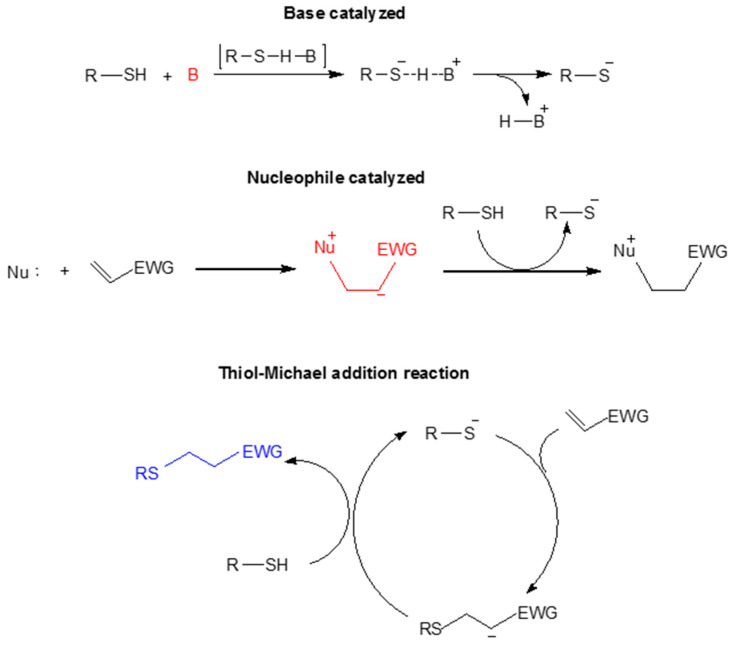
Schematic thiol–Michael reaction mechanism. (EWG represents an electron withdrawing group). In blue Michael thiol-adduct and in red initiating species are indicated.

Cross-linked polymer networks prepared by the thiol-Michael addition reaction via step-growth polymerization are the most common strategy; these have been used intensively in recent years to obtain this type of material. This reaction can be base- or nucleophile-catalyzed. The base-catalyzed Michael reaction proceeds via hydrogen abstraction from the thiol to yield a thiolate, and it is fundamentally controlled by the strength and concentration of the base and the thiol p*K*a. The nucleophilic path is favored when a strong nucleophile is used, such as a phosphine, which attacks the Michael acceptor and results in the formation of a zwitterion that acts as the initiating species [78,85,86]. This mechanism is depicted in Scheme 7.

In the recent literature, many methods of thermoset preparation by means of Michael additions have been described. Among these, an interesting approach was documented by Bowman et al. [79]. They prepared a composite material composed of two polymer networks formed from two consecutive thiol-Michael reactions using a unique nucleophilic initiator but involving monomers with different reactivities. A vinyl sulfone and an acrylate were used as Michael acceptors, whereas a mercaptoacetate and a mercaptopropionate were used as Michael donors. The first network was formed at room temperature by reaction between the mercaptoacetate and the vinyl sulfone, and the second one at 90 °C by reaction between the acrylate and the mercaptopropionate. The final material showed two glass transitions associated with the two different networks formed, and exhibited triple shape memory behavior. The same group showed the high reactivity and selectivity of vinyl sulfones over acrylates via nucleophile-catalyzed thiol-Michael addition in ternary formulations; this was used to control the gelation behavior and prepare polymers with distinct functionalities and final properties. Moreover, it was shown that if acrylates and vinyl sulfones with equal functionalities are used, neat thiol-vinyl sulfone networks exhibit much higher glass transition temperatures than their thiol-acrylate counterparts [87]. Replacing acrylates by vinyl sulfone monomers can be a robust strategy to avoid the inherent high rotational flexibility around the thioether linkages which results in low glass transition temperatures of thiol-acrylate networks. Two drawbacks must be considered: the limited number of commercial vinyl sulfones available and the difficult of controlling of reactivity. The latter is highly influenced by the catalyst, the preparation of the formulation and the presence of protic species other than thiols in the reaction mixture. It has been demonstrated that the presence of methanesulfonic acid completely stopped the reaction between a mixture of thiols and ethyl vinyl sulfone in the presence of a phosphine as an organocatalyst, whereas the presence of water only decreased the reaction rate. For this system it has been proved that the nucleophilic catalyst should not be added to the vinyl monomer before the thiol-Michael reaction proceeds, since the zwitterion, which is formed by the attack of the nucleophile to an electron-deficient vinyl group, could start side reactions and cause the Michael reaction to lose its click nature [88].

The mechanical properties of a thiol-Michael network can be enhanced using maleimide as the Michael acceptor instead of acrylates [89,90]. Voit et al. prepared solvent-free thiol-maleimide networks exhibiting glass transition at temperatures around 80 °C, higher than other thiol-click systems such as thiol-acrylate and thiol-epoxy [89]. However, the preparation of thiol-maleimide networks is relatively complex due to the inherent high reactivity of this curing process.

As mentioned, networks formed by thiol-Michael addition reactions have relatively low glass transition temperatures and crosslinking densities due to the high content of flexible thioether linkages and to the low effective monomer functionality, both of which limit their use in potential applications requiring high $T_{g}$ and hardness. In comparison with a chain-grown network, such as the obtained by radical polymerization, acrylates act with half their functionality in Michael additions. In consequence it is possible to enhance thiol-Michael network properties by combining these networks with those obtained by radical acrylate polymerization, despite the loss of the click nature of the process. With this reasoning, Bowman et al. prepared a new family of dual networks from off-stoichiometric thiol-acrylate formulations. The first stage was a self-limiting thiol-Michael reaction and the second was a UV-induced polymerization of the excess of acrylate groups. The obtained materials show a wide range of properties, depending on the functionality and feed ratio of the monomers, which makes them optimal for multiple applications such as shape memory materials, 3D printing and optical materials [24]. The final properties of this kind of thermoset can be easily tuned by replacing the acrylate excess with methacrylate monomers that barely react during Michael addition reaction [81,83], due the inductive effect and steric hindrance of the methyl group in the methacrylate. Only in the presence of higher amounts of tertiary amines or a superbase are methacrylates able to react with thiols via Michael addition [83,91].

A similar strategy was used previously by Moszner et al. [92,93], and recently, by Ramis et al. [82] in order to obtain highly crosslinked networks via a carbon-Michael reaction. In these cases, the first stage was a self-limiting acetoacetate-acrylate Michael addition and the second was radical homopolymerization of the added methacrylate or the excess of acrylates. In a ternary acetoacetate-acrylate-methacrylate system, the $T_{g}$ increases from 25 °C (neat acetoacetate-acrylate mixture) to 119 °C (neat methacrylate) when the content of methacrylate is increased [82]. Another interesting advantage of an acetoacetate over a thiol as a Michael donor is that the acetoacetate contains two acidic protons which are potentially reactive as Michael donors. The glass transition temperature of a stoichiometric triacetoacetate/triacrylate thermoset changes from 24 °C to 76 °C if the functionality is six, rather than three [82].

Recently, a novel set of dual-curable multiacetoacetate-multiacrylate-divinyl sulfone ternary materials with versatile properties was prepared via two consecutive carbon-Michael additions, confirming again the selectivity of vinyl sulfones over acrylates. In contrast to common dual-curing systems, the first stage polymer herein consists of a densely crosslinked, high glass transition temperature network as a result of a base-catalyzed multiacetoacetate-divinyl sulfone Michael addition. A more flexible secondary network forms after the base-catalyzed Michael addition of remaining multiacetoacetate to multiacrylate [80].

Thermal and mechanical properties of otherwise flexible and soft thiol-Michael networks can be significantly increased by the addition of acetoacetate monomers. In one such attempt, thiol-acetoacetate-acrylate ternary dual-curing thermosets were prepared by a sequential process consisting of thiol-Michael addition to acrylates at room temperature, followed by Michael addition of acetoacetates to acrylates at a moderately elevated temperature (e.g., 80 °C). The curing sequence could easily be controlled with the help of the different acidities of the protons on thiol and acetoacetate groups, with the favorable p*K*a of the base being used as catalyst and the self-limiting character of Michael additions. In general, the stability of the monomer mixture and of the intermediate materials, obtained after thiol-Michael addition, can be established using a photobase generator. Among these, tetraphenyl borate salts have proved to be useful since they can be activated on demand by UV light or thermally. Moreover, their activity, once the base is released, depends exclusively on the p*K*_a_ of the base used [94].

The use of amines instead of thiols to obtain Michael thermosets with a wide range of improved properties introduces some advantages [81,95,96]: (a) since amines can act as both nucleophiles and bases, an additional base may not be necessary in aza-Michael additions; (b) the tertiary amine formed during the reaction acts as a catalyst, accelerating the curing process; (c) the presence of amines in the reaction media overcomes the intrinsic oxygen inhibition of acrylate free radical polymerization, resulting in completely cured thermosets in dual amine/acrylate formulations with an excess of acrylate; (d) the existence of a large number of commercial amines available with different functionalities and structures; (e) primary amines have twice the functionality of thiols in Michael addition reactions, and (e) the different reactivity of primary and secondary amines makes it possible, with adequate selection of amines and acrylates, to obtain thermosets prepared via two sequential aza-Michael additions. Allonas et al. developed an elegant synthetic strategy based on aza-Michael addition and radical photopolymerization to generate a polymeric network via three time-controlled steps. Furthermore, it is possible to interpose a radical photopolymerization reaction between two consecutive aza-Michael reactions, thereby enhancing the cross-link density [30].

Although oxa-Michael is not usually used in the preparation of click thermosets, thanks to the amount of commercially available alcohols and acrylates, it stands out as a useful methodology that must be explored more in detail. Recently, a diene rubber functionalized with hydroxyl pendant groups was effectively cured (vulcanized) by various acrylates based on the oxa-Michael reaction without the use of any additional additives. The final properties of diene rubbers are easily controlled by hydroxyl content and by the content and functionality of the acrylates [97].

## 5. Preparation of Thermosets by Addition of Thiol Monomers on Unsaturated Compounds

**Scheme 8:**
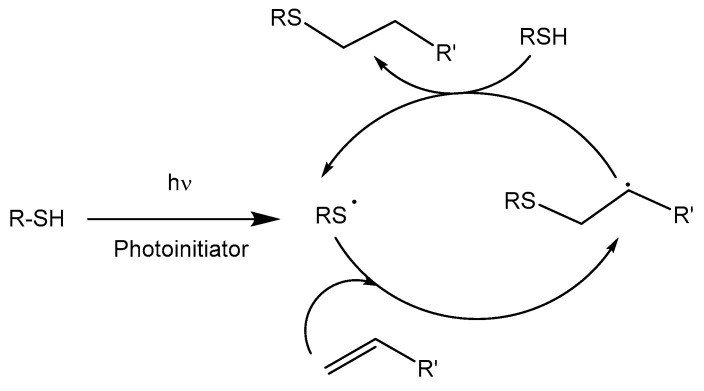
Mechanism of the thiol-ene reaction.

Thiol-ene and thiol-yne reactions constitute a type of radical photopolymerization initiated by UV irradiation with some advantages over traditional vinyl photopolymerization reactions [98]. One such advantage is that this process is not inhibited by the presence of oxygen; therefore, it can be performed in an air atmosphere and possesses spatial and temporal control enabled by UV-initiation. Moreover, this is a stepwise process that usually leads to highly homogeneous networks if one starts from monomers with the adequate functionality. The mechanism consists of the alternation between thiol radical propagation through the *-ene* functional group and the chain-transfer reaction, which involves the abstraction of hydrogen radical from the thiol by the carbon radical, usually in the presence of a photoinitiator, following the mechanism depicted in Scheme 8. The addition of a thiyl radical to the vinylic unit proceeds with an anti-Markovnikov regioselectivity [99]. It is also possible to perform the reaction thermally by using a radical thermal initiator, such as α,α’-azobis(isobutyronitrile), AIBN [100]. To avoid vitrification, the photoirradiation process should be performed at high temperatures.

**Scheme 9:**
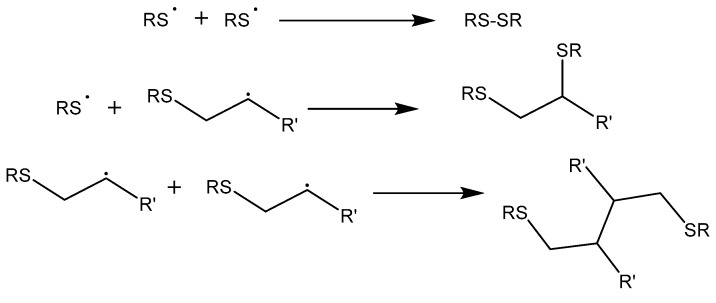
Possible termination reaction during thiol-ene process.

Although this reaction has been considered as a click process, some undesired by-reactions can occur, as depicted in Scheme 9, which limit the conversion. As shown, the possibility of the recombination of radicals forming S–S, S–C or C–C bonds will stop the thiol-ene process. Another possibility is that the carbon radical formed can initiate the homopolymerization of the vinylic units, limiting the formation of thioethers. In case of acrylates, the homopolymerization reaction is a competitive process.

The reactivity of the *-ene* moiety in a thiol-ene reaction depends on its degree of substitution. Highly substituted alkenes are less reactive than monosubstituted ones, probably due to steric effects. In addition, alkene reactivity decreases with decreasing electron density of the carbon–carbon double bond. In reference to the effect of the thiol structure, it has been reported that thiols based on propionate esters and glycolate esters are more reactive because of a weakening of the sulfur–hydrogen bond by hydrogen bonding of the SH groups with the carbonyl ester [98].

It is noteworthy, as discussed in Section 2, that the polycondensation mechanism adopted in this reaction leads to a homogenous distribution of crosslinking points in the whole material and to a great adhesion to a wide variety of substrates. This is because of the low internal stress in the network, since a great extent of the reaction occurs before gelation, when the mixture is still a viscous liquid. In addition, the thioether linkages formed during the addition of the thiol to the *-ene* compound are flexible, thus promoting adhesion [101].

The main drawbacks of thiol-ene chemistry are the strong odor of thiols, limited shelf life, low glass transition temperatures and poor mechanical properties, such as hardness, mainly due to the structure of the thiols used [102]. The use of thiolactones has been proposed to circumvent some of these problems [103]. In the preparation of thermosets, it should also be considered that the conversion of a vinyl to a single thioether implies that the vinyl group is acting as monofunctional, and therefore, the ultimate cross-link densities of these materials can be rather low. Higher cross-link density can be achieved by increasing monomer functionality.

The thiol-ene reaction has been applied with terpenes as starting resins for 3D printing technologies [104] and, in general, to the preparation of thermosetting materials from renewable materials such as lignin [105,106], eugenol [107,108], furan derivatives [109], allylated aminoacids [110] and resveratrol [111]. Keratin, which is a natural polymer found in hair and nails, contains thiols in its cysteine and methionine structures; as such, it has been used as a renewable natural reinforcing agent for photocured thiol-ene coatings [112].

The viscoelastic loss factor of thiol-ene amorphous thermosets is greater than most polymers in the vicinity of the glass transition. These thermosets could be beneficial for personal protective equipment or other applications requiring high damping of mechanical energy, because of the reduced $T_{g}$s of this class of thermosets [113]. To develop such thiol-ene thermosets with tunable $T_{g}$s, a mixture of two different thiols with different functionalities was employed. This strategy aimed to maintain desirable characteristics such as rapid kinetics of formation and high, narrow tan δ values in the glass transition region [114]. A minimal broadening of the glass transition region was observed, which implies the retention of the energy absorbing capabilities of the material with a $T_{g}$ that could be accurately predicted by the Fox equation. Bowman et al. prepared a hybrid organic/inorganic thiol-ene based photopolymerized network using POSS derivatives with thiol groups obtained by sol-gel process. The materials obtained showed high $T_{g}$ and relaxed modulus, increasing with the amount of inorganic content in the material because of the multifunctionality of the POSS structures [115]. Thiol-ene networks with reactive surfaces were prepared by Trey et al. [116] starting from an allyl functionalized, Boltorn-type hyperbranched polyester. Because of the high functionality of the hyperbranched polymers, allyl homopolymerization could occur, but the residual thiol groups that remained unreacted after photopolymerization allowed postfilm formation reactions to take place, affording overcoats with a better interfacial adhesion.

**Scheme 10:**
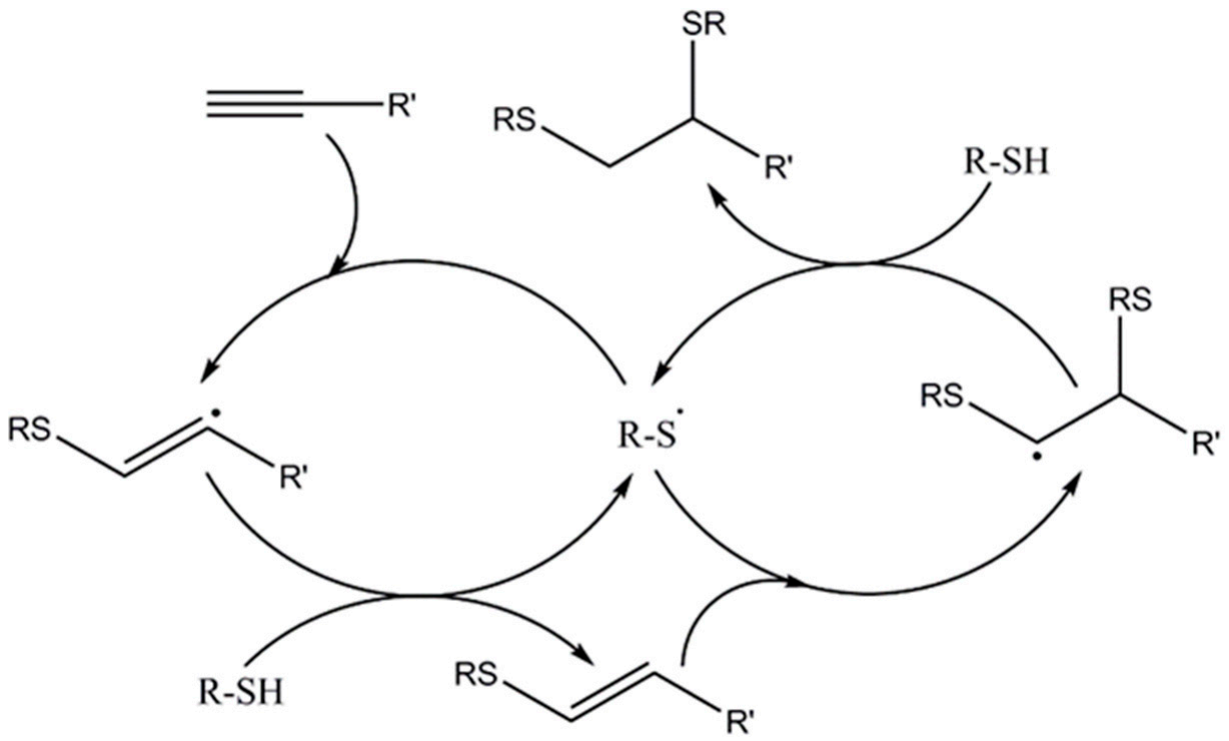
Mechanism of the thiol-yne reaction.

Similar to thiol-ene, a thiol-yne reaction is a radical-mediated click reaction which takes place in a two-step process: the addition of thiol to the carbon-carbon triple bond to yield an intermediate vinyl thioether, followed by a thiol-ene reaction with the remaining thiol, which leads to 1,2 double-addition species [117]. The proposed mechanism is depicted in Scheme 10.

From a kinetics point of view, the rate of alkyne disappearance is equal to the rate of thiol loss and vinyl creation. The formed vinyl sulfides are consumed immediately upon creation, and therefore, alkyne presents a functionality of two in thiol-yne step-growth polymerizations. However, when the alkyne is in excess, the accumulation of vinyl sulfide is more pronounced than in stoichiometric reactions. During photopolymerization, the rate of thiol addition to the vinyl sulfide formed was observed to be ∼3 times that of the thiol addition to the alkyne. Bowman’s group demonstrated that thiol-yne materials have a higher crosslink densities, glass transition temperatures and moduli compared to a similar thiol-ene network; therefore, they possess better characteristics as structural materials [118].

## 6. Poly(thiourethane) Networks by Thiol-Isocyanate Reaction

**Scheme 11:**
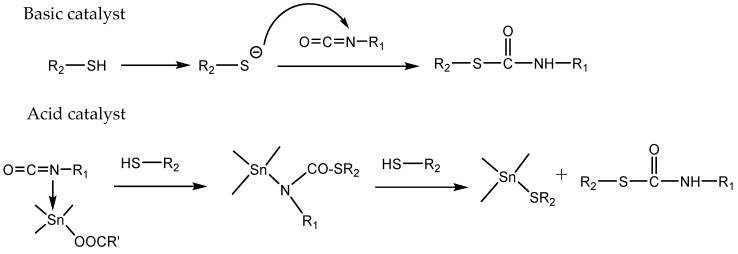
Proposed mechanisms of the formation of poly(thiourethane) in basic and acidic conditions.

The base-catalyzed thiol-isocyanate reaction has been included in the group of “click” type reactions [119,120] because of the high reactivity of thiols towards isocyanates in these conditions, yielding thiourethanes, also named thiocarbamates, in high-to-quantitative yields (see Scheme 1) without the formation of unexpected structures. This reaction can also be catalyzed by Lewis acids, usually with Tin as a catalyst, but in this case the click characteristics, seems to be lost. Scheme 11 presents the proposed mechanism in acid and basic media.

The most common basic catalysts involved in the thiol-isocyanate polycondensation are tertiary amines such as triethylamine (TEA) or amidine compounds such as 1,5-diazabicyclo[4.3.0]non-5-ene (DBN) or 1,8-diazabicyclo[5.4.0]undec-7-ene (DBU) [121]; however, in the preparation of thermosets, the reaction is too fast, with short pot-lives that enormously complicate their application [122]. The fast reaction is due to the low pKa of thiols and their easy deprotonation in the presence of a base. To prevent these drawbacks, latent amine initiators have been proposed, activated by irradiation [123] or heat [124].

The fast thiol-isocyanate reaction has been exploited to develop an original autonomous self-healing system for epoxy thermosets. The addition of separate capsules containing either isocyanates or thiols facilitates the extrinsic self-healing of epoxy, during which the amine groups present in the matrix act as a basic catalyst [125].

Crosslinked poly(thiourethane)s have been widely used in optical materials thanks to their high refractive indexes [126]. From commercially available diisocyanates and tri- and tetra- thiols of different structure, and using dibutyltin dichloride as the catalyst, a series of thermosets with excellent homogeneity, optical characteristics and good mechanical performance were obtained. In another interesting study on this type of material, the phenomenological behavior of the network formation and its effect on the thermomechanical properties of the crosslinked material were investigated in detail [127].

Thermosetting poly(thiourethane)s have also been prepared from renewable resources [128] using dibutyltin diacetate as the catalyst. The coatings showed good adhesion to aluminum and had high gloss. However, they exhibited low tensile strength, moduli and chemical resistance due to the flexibility of the fatty acid chain.

An interesting feature of these poly(thiourethane) networks is that they behave like vitrimers. Torkelson’s group reported the vitrimeric behavior of poly(urethane) elastomers prepared from a mixture of PPh_3_ and DBN with [129] and documented that the aromatic isocyanate polymeric derivatives follow associative and dissociative reversible pathways, depending on whether an excess or a stoichiometric amount of thiol has been added to the formulation. More recently, our research group reported an associative behavior of aliphatic poly(thiourethane) networks prepared in the presence of dibutyltin dilaurate (DBTDL). The relaxation was faster when the amount of catalyst increased [130]. We also reported the possibility of reshaping or recycling crosslinked poly(thiourethane)s with $T_{g}$s of around 130 °C [131].

## 7. Preparation of Thermosets by Azide-Yne Polymerization

The first documented click reaction was the azide-yne, a 1,3-dipolar cycloaddition reaction that leads to the formation of 1,2,3-triazole moieties, established by Huisgen [132] (see Scheme 1). It is usually performed at high temperatures and the -yne groups can be terminal or internal. This is the most widely used click reaction, together with some of its variants [133].

There is a huge number of materials based on the use of Huisgen thermal azide–alkyne cycloaddition. For instance, it has been applied to the preparation of silicon networks taking advantage of the higher reactivity of carbonyl-functionalized alkynes. The reaction continues at room temperature for several days [134]. These reactions have also been applied to the preparation of highly-crosslinked hybrid materials from azidated POSS and aliphatic polyesters with internal triple bonds [135,136]. The thermal and mechanical properties of hybrid networks were clearly enhanced by increasing the POSS-N_3_ concentration.

A variation of this reaction is copper(I) catalyzed, as reported independently by Meldal [137] and Sharpless [138] in 2002, also known as Copper(I)-catalyzed Azide-Alkyne Cycloaddition (CuAAC). While Huisgen’s reaction leads to a mixture of 1,4 and 1,5-disubstitution products, the CuAAC reaction of terminal alkynes is completely regioselective in the formation of the 1,4-disubstituted triazoles [139]. This reaction usually needs the addition of a reduction agent to convert Cu(II) to Cu(I), being the most extended sodium ascorbate and an amine ligand, which enhances the reaction rate. The simplicity of the CuAAC reaction, together with its high yield, fast kinetics and orthogonal reactivity has moved many researchers to include this click reaction in the preparation of a great variety of advanced polymers.

The step-growth nature of the CuAAC reaction applied in the curing of multifunctional azide-yne formulations results in uniform network formation, which affords shape memory thermosets with a shape-fixity and recovery ratio of at least 99% with sharp transitions [140].

In a different application, a glycidyl azide polymer (GAP) was cured through click chemistry by the reaction of the azide group with bispropargyl succinate (BPS) through a 1,3-dipolar cycloaddition reaction to form a 1,2,3-triazole network. The higher heat of exothermic decomposition of triazole adduct (418 kJ/mol) against that of azide (317 kJ/mol) and better mechanical properties of the GAP-triazole render it a better propellant binder than a previously prepared GAP-urethane system [141].

Although extensively used, this reaction has some drawbacks. Firstly, it usually lacks spatial and temporal control in the initiation step, which could complicate the application of such materials in some technologies, such as coatings [142]. Secondly, the obtained polytriazole networks contain metallic residues which are generally detrimental to electronic and optical properties. Light emissions of conjugated polymers, for example, can be quenched by metallic traps. Finally, transition-metal catalysts are expensive. The minimal use of metallic catalysts to establish true control of the process and the development of metal-free polymerization systems is thus required [133].

The CuAAC reaction can also be performed using photoirradiation. An air-stable Cu(II) complex such as CuCl_2_ can be used with or without a photosensitive reducing agent in the presence of an alkylamine ligand such as *N,N,N,N,N*-pentamethyl diethylenetriamine (PMDETA) and light, which results in the formation of a Cu(I) complex which catalyzes the azide-yne reaction [143]. This system makes it possible to spatially and temporally control the polymerization procedure [144]. A photochemically-induced, Cu (I)-catalyzed azide−alkyne cycloaddition click reaction (CuAAC) was applied to the preparation of thermoset networks from soybean oils as a renewable resource. Clickable azide and alkyne functionalities were introduced into epoxidized soybean oils by simultaneous ring-opening reactions between the epoxide group of soybean oils and sodium azide and propargyl alcohol, respectively, and then crosslinked by photoinduced CuAAC [145].

The CuAAC reaction has also been applied to the formation of step-growth bulk photopolymer networks, where the photopolymerization generates triazole adducts that are thermally, chemically, and mechanically stable [146]. Triazole structures, especially in a densely cross-linked network, substantially enhance the mechanical stiffness and strength of these materials during the polymerization.

In their paper, Tasdelen et al. described the preparation of antibacterial thermosets by combining simultaneous photoinduced electron transfer and photoinduced CuAAC processes [147]. The radicals, photochemically generated from DMPA, not only help to form Ag^0^ nanoparticles from Ag^+^ cation, but also reduce Cu (II) into Cu (I), which catalyze the CuAAC of multifunctional azide and alkyne compounds. The antibacterial thermosets could be prepared at room temperature and were able to inhibit the growth of both gram-negative and -positive bacteria, the latter thanks to the formed Ag nanoparticles whose antibacterial properties are well known.

In another work, phenolic crosslinked epoxy thermosets were obtained through an azide-ine click reaction [148]. Propargylated novolac and bis azido hydroxy propylether bisphenol A were synthesized and cocured by click reaction under catalyzed and uncatalyzed conditions to form triazole networks. The cure reaction which occurs around 130 °C decreased to 74 °C on catalysis by Cu_2_l_2_.

Larger molecules can also be employed in a CuAAC-type crosslinking process. Hyperbranched polyphenylene (hbPPh) containing acetylenic groups was crosslinked with a small molecule crosslinker 1,3,5-tris(azidomethyl)benzene (TAMB). It was shown that the crosslinking of hbPPh/TAMB (9:1) mixtures makes it possible to fabricate a film either thermally or photochemically by making use of fundamentally different reaction mechanisms [149].

It has been also reported that multivalent azides and alkynes, when deposited between surfaces containing metallic copper, are crosslinked by Cu(I)-mediated triazole formation to promote strong adhesion of one surface to the other [150,151]. Other studies by lap shear tests showed that crosslinkable polytriazole resins have much higher adhesion strength than bisphenol A epoxy adhesive to copper, iron and aluminum at temperatures higher than 150 °C in humid environments [152].

## 8. Preparation of Thermosets by Diels-Alder Cycloaddition

The [4+2] cycloaddition reaction proposed in 1928 by Diels and Alder is one of the most popular reactions in organic synthesis. In this reaction, an electron-rich diene and an electron-poor dienophile react without the need of catalyst (see Scheme 1). This reaction occurs at moderate temperatures and has a reverse process (retro-Diels-Alder) at elevated temperatures. This reversibility, triggered under different conditions, facilitates not only the reworkability of thermosetting materials, but also the possibility of special applications such as remendable materials [153,154] and other smart technologies [155].

Diels-Alder reaction (D-A) was also used in the preparation of thermosets by modification of polystyrene with maleimide groups [156].

The reversible D-A process has made it possible to prepare new types of recyclable or self-healing thermosets, such as epoxy resins [157,158], polyurethanes [159], polyamides [160] or polybenzoxazines [161], and even vulcanized rubber [162]. However, the most representative work in this area was published by Wuld et al. [163,164]. Starting from maleimides and furan derivatives with a high functionality, a network is formed. The material was a tough solid at room temperature with mechanical properties similar to those of commercial epoxies. At temperatures above 120 °C, approximately 30% of the Diels-Alder adducts disconnected but then reconnected again upon cooling. This process can be repeated several times with high remendability efficiency. There are a great number of remendable thermosets based on this strategy [165,166].

**Scheme 12:**
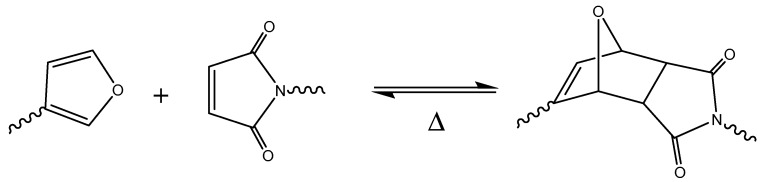
Representation of the reversibility of furan-maleimide adducts by Diels-Alder process.

Some of the so-called covalent adaptable networks (CANs)—i.e., are cross-linked polymers in which the crosslinking strands within the polymer material can undergo reversible rearrangement reactions—have D-A adducts in their structure. The D-A rearrangements provide a microscopic mechanism for achieving macroscopic flow and stress relaxation in thermosetting polymers for which this was previously impossible [167,168,169]. The reversible process is depicted in Scheme 12.

**Scheme 13:**
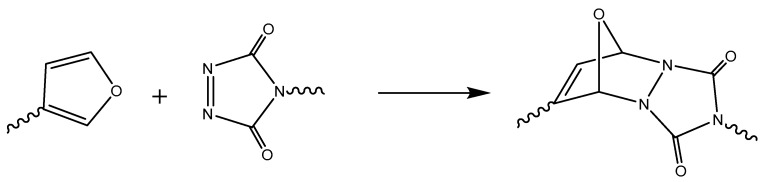
Diels-Alder reaction with 1,2,4-triazoline-3,5-dione as dienophile.

Including an adequate selection of dienes and dienophiles, a lot of studies have been published with different types of thermosetting materials. As a diene, furan derivatives have been widely used, e.g., in the work by Gandini et al. [170,171,172]. Other dienes such as cyclopentadiene [173] or anthracene [174,175] -functionalized compounds have also been reported. As dienophiles, maleimides are the most commonly employed [176,177], although Du Prez et al. proposed the use of 1,2,4-triazoline-3,5-dione (TAD). The reaction with TAD is fast at room temperature, but the reversibility is lost [178]. However, by changing the diene structure, reversibility can be recovered. Scheme 13 exemplifies a reaction involving TAD and a furan derivative. 

Four-arm star-shaped end-functionalized PCLs have been modified to introduce furan, maleimide and anthracene final groups that are able to undergo Diels–Alder cycloaddition with maleimides to produce thermosets [174]. The anthracene-based network exhibits much faster curing kinetics than the furan-based network, which also has a lower crosslinking rate, due to the occurrence of a retro Diels–Alder reaction during curing at 60 °C. The materials obtained are partially crystalline and their shape memory characteristics have been studied and related to the reversibility of the network structure.

Modified novolac epoxy resins with furan pendant groups were prepared from novolac epoxy resin and furfuryl alcohol and then crosslinked by bifunctional maleimide via Diels-Alder reaction to obtain thermally reversible and self-healing novolac epoxy resins [179].

Thermosets were also obtained by crosslinking linear polymers with furan and maleimide functionalized dangling chains [180] or with furan by adding bismaleimide as the crosslinker [181,182]. All of these materials showed good self-healing capabilities.

It is worth mentioning that biobased materials, namely vegetable oils [183,184] or tannins [185] have been also prepared by D-A reaction.

**Scheme 14:**
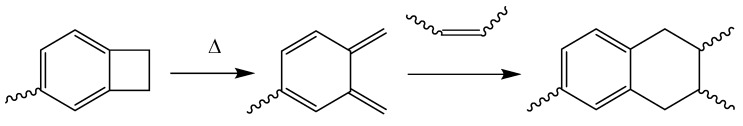
Reaction D-A mechanism between a benzocyclobutene and a vinyl group.

Benzocyclobutene-functionalized monomers are able to react at elevated temperatures (up to nearly 200 °C) with dienophiles to form D-A adducts [186,187,188]. The strained four-membered ring on the benzocyclobutene group opens to produce a reactive intermediate, o-quinodimethane, as represented in Scheme 14.

## 9. Combination of Two or More Click Reaction: Dual Curing

**Figure 6:**
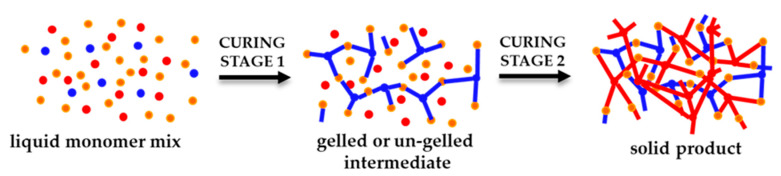
Schematic representation of a sequential dual-curing process.

Among the many thermoset preparation strategies, dual-curing stands out as an effective and versatile method. It is based on two polymerization processes carried out either concurrently or sequentially, as depicted in Figure 6.

A deep understanding of the underlying reaction mechanisms is crucial to obtain tailor-made materials through curing sequences with well-established kinetics. The material properties at different stages of curing are governed by the choice of monomer. Moreover, the utilization of click procedures contributes greatly to sustainability and efficiency of the method. Several sequential click curing systems have been studied and characterized to date using a variety of techniques, such as calorimetry or spectroscopy. Also, some other dual-curing systems have also been developed simply in order to improve the properties of otherwise single-cure thermosets.

The sequentiality in curing provides immense flexibility in the industrial processing of these materials; a chemically stable intermediate material can be stored indefinitely and manipulated whenever desired to process it further. This sequentiality and stability is made possible either by the intrinsic chemistry of the selected monomers, or by employing latent catalysts, which, upon stimulation by heat or light, liberate the active species to initiate curing. This way, complete temporal control of the curing process is facilitated.

In some dual-curing systems, some click polymerizations such as stepwise Michael additions are combined with chain-wise homopolymerizations such as acrylate photopolymerization. In such systems, the stepwise polymerization, at the first curing stage, imparts advantages such as network homogeneity, high gel point conversion, and low curing shrinkage to the material. Later, the secondary chain-wise polymerization stage increases the material’s crosslinking density, glass transition temperature and hardness, thereby improving its mechanical properties significantly. Without regard to the utilized curing process, all dual-cured final materials exhibit improved thermal and mechanical properties in comparison to the intermediate materials. These dual-curable materials can be used in a wide array of value-added applications such as high-performance adhesives, shape memory materials, vitrimers, and 3D printing resins (see Section 10).

One of the earliest dual-click procedures was described by Chan et al., whereby a sequential curing process was employed using thiol-ene and thiol-yne reactions as first and second stages, respectively [189]. Highly functional materials were prepared under benign conditions, i.e., at ambient temperature and under air atmosphere. The thiol-ene stage was extremely fast and quantitative, thanks to the use of a nucleophilic catalyst, namely dimethylphenylphosphine (Me_2_PPh). This Michael type reaction was followed by a radical-mediated thiol-yne reaction, which was orthogonal to the thiol-ene stage. This radical thiol-yne reaction quickly and quantitatively yielded the 1,2-addition product.

**Scheme 15:**
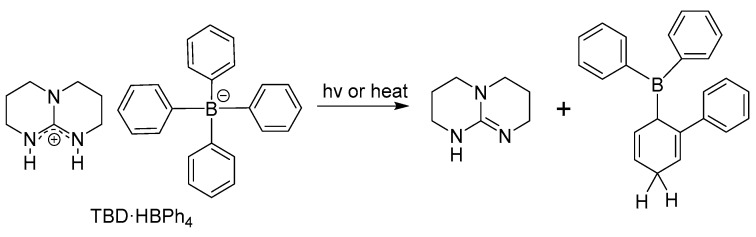
Activation of a tetraphenyl borate salt photobase generator.

Versatile thiol chemistry was utilized by another group of researchers to design a thiol-isocyanate-ene dual-curing system [190]. In their system, the two reactions, namely thiol-isocyanate and thiol-ene reactions (Scheme 1d,c respectively), took place either simultaneously or sequentially, depending on the reaction trigger employed; a thermally-active base catalyst triethyl amine (TEA) led to sequential reactions, whereas simultaneous curing was achieved when a photolatent base catalyst (tributylamine tetraphenylborate salt, TBA HBPh_4_) was used, from which TBA was liberated upon light exposure. As the photoinitiator for the thiol-ene reaction, 2,2-dimethoxy-2-phenyl acetophenone (DMPA) was used. Both reactions proceeded quantitatively. Processing flexibility was achieved by the control of curing sequence which allowed tailor-made fabrication of photocurable thin films or thick materials. Furthermore, the incorporation of thiourethane groups in thiol-ene networks improves the physical and mechanical properties thanks to hydrogen bonding. It has been documented that the $T_{g}$ of these thiourethane/thiol-ene hybrid networks increases from −5 to 35 °C as the thiourethane content increases. The same group also described a thiol-isocyanate-acrylate system wherein the thiol-ene reaction was replaced by a thiol-acrylate Michael-type reaction (See Scheme 1b) followed by the photoinitiated homopolymerization of remaining acrylate groups [191]. They showed that both reactions were quantitative, and that the thiol-isocyanate coupling was completed in 1 min, while the thiol-acrylate Michael addition took 10 min to complete, making it a sequential process. The material properties were highly tunable, with $T_{g}$ ranging from 14 to 100 °C, according to the composition. Fast thiol-isocyanate coupling was also used as a first curing stage in a click-based system proposed by Gamardella et al. in which it preceded a thiol-epoxy reaction [192]. Taking advantage of the vast difference in the rates of the two reactions, the two curing stages could be initiated at different temperatures, thereby ensuring sequentiality. The materials showed desirable, narrow glass transition properties for smart applications. A simultaneous dual-curing process was also documented by Perrot et al. for a similar thiol-isocyanate-epoxy ternary system [193]. They used a photoinitiated latent catalyst, namely a tetraphenylborate salt of 1,5,7-triazabicyclo[4.4.0]dec-5-ene (TBD). As depicted in Scheme 15, upon being irradiated or subjected to heat, the tetraphenylborate salt underwent rearrangement and liberated the active base.

They showed that the thiourethane network enhanced the thiol-epoxy properties through an increased rate by hydrogen bonding. The versatility of material properties according to the choice of monomer was demonstrated.

As for interesting applications of dual-curing philosophy, the Bowman group came up with an elegant process for preparing holograms [194]. They first prepared loosely cross-linked soft elastomeric substrates through a thiol-acrylate Michael addition. These first stage “intermediate” materials contained uncured -*ene* and excess of thiol groups which underwent UV-initiated click thiol-ene photopolymerization, allowing holographic images to be recorded onto them. The final materials were graded rainbow holograms with high $T_{g}$, high modulus, diffraction efficiencies of up to 82%, and refractive index modulation of 0.004.

**Figure 7:**
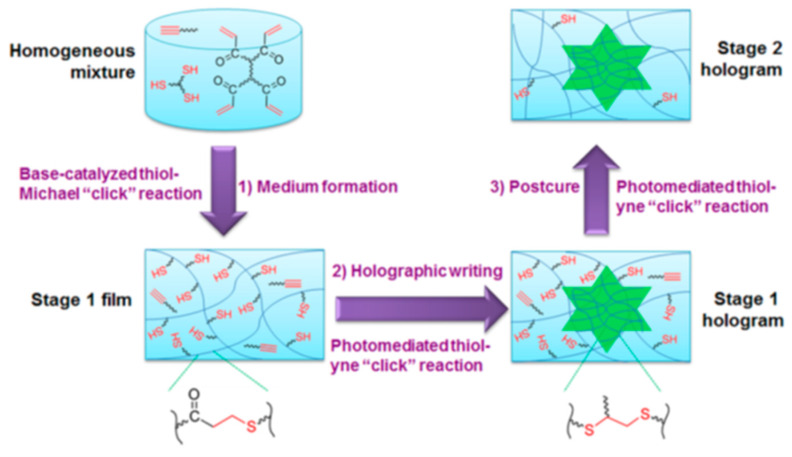
A thiol-acrylate-yne ternary system for holographic patterning. Reproduced with permission from [195]. Copyright: 2014, American Chemical Society.

Another publication came from the same laboratory describing an image patterning technique based on a different dual-curing procedure, as depicted in Figure 7 [195]. This time, the base catalyzed thiol-Michael addition was followed by a radical-initiated thiol-yne click reaction. The authors utilized a custom-synthesized, multifunctional alkyne monomer that allowed high index gradients to be recorded. Final materials with refractive index modulations of 0.0036, diffraction efficiencies up to 96%, good oxygen resistance, and volume shrinkage down to 1.1% were obtained; these were intended for applications such as high-density data storage, image patterning and anticounterfeiting. The procedure also involves a postcuring step to achieve the best possible material properties.

Since thiols can also cure epoxy resins, a thiol-epoxy reaction can be carried out as a second curing step, given that an excess of thiols remains from the first curing stage; a covalently-bound interpenetrating polymer network would be obtained as a result. Following this philosophy, Guzmán et al. designed a thiol-ene/thiol-epoxy dual-curing system wherein a photoinitiated thiol-ene reaction took place at the first stage, and an epoxy-thiol reaction followed, initiated by an encapsulated (latent) imidazole catalyst [196]. The active imidazole was liberated from its encapsulation once the temperature was raised. As in all sequentially dual-curable thermoset formulations, the intermediate materials had a certain stability, and the material properties at both curing stages were clearly defined and tailorable by changing the initial monomer mix. The researchers used the same dual-curable chemistry later to design bio-based thermosets based on eugenol [197]. A synthetic allyl glycidyl derivative from eugenol was dual-cured using a thiol derivative, which was also based on eugenol, through a photoinitiated thiol-ene reaction, followed by thermal thiol-epoxy curing. As expected, these dual-cured materials had better thermal and mechanical properties compared to the neat thiol-ene thermosets prepared from similar allyls derived from eugenol. Before this publication, Dai et al. described the use of a UV/thermal dual-curing process to prepare eugenol-based antibacterial films. As the UV-initiated thiol-ene reaction failed to proceed to completion, a thermal curing step was added to ensure that the depletion of all remaining monomers had occurred, thereby yielding films with better properties [198]. A similar system was proposed later by Shibata et al., wherein a eugenol-derived epoxide was dual-cured through a photoinitiated thiol-ene reaction, followed by thermal thiol-epoxy reaction. By adding a maleimide compound, a thiol-maleimide reaction could also be carried out concomitantly to thiol-epoxy, which resulted in materials with higher $T_{g}$, tensile strength and modulus [199].

Another thiol-ene/thiol-epoxy photo/thermal dual-curing system was documented by Acebo et al. [200]. They added a custom-synthesized hyperbranched poly(ethyleneimine) with allyl terminal groups into a thiol-epoxy mixture in such a way that the thiol:ene:epoxy stoichiometry was conserved. The first curing stage was a photo-initiated thiol-ene reaction, followed by an imidazole catalyzed thiol-epoxy reaction. Since the allyl-functionalized hyperbranched had tertiary amine groups in its structure, the thiol-epoxy reaction started prematurely during the UV treatment of the formulations. Nevertheless, the addition of a basic catalyst (imidazole) was still necessary to complete the reaction in a thermal oven in a practical time frame. The poly(ethyleneimine) macromonomer imparted flexibility and, thus, lower $T_{g}$ to the resulting network, but increased its rubbery modulus due to a higher density of cross-links. Some researchers from this group later carried out a dielectric thermal analysis (DETA) of the same materials and characterized their complex relaxation behavior [201]. Similarly, a thiol-yne reaction was combined with thiol-epoxy taking propargyl-functionalized hyperbranched polyethyleneimine. The double addition of thiol groups to the alkyne moieties allowed them to reach higher glass transition temperatures in the thermosets, according to their higher crosslinking densities [202].

In another publication, a dual-cured thiol-ene/epoxy system was described [203], wherein an allyl functional hyperbranched polyester was first cured with a trithiol compound via a photoinitiated thiol-ene click reaction to yield a flexible thioether network. This, in turn, formed trialkylsulfonium salts through a reaction with the epoxy groups. Upon heating to temperatures above 100 °C, these salts efficiently initiated cationic epoxy homopolymerization and formed the final network, with significantly higher $T_{g}$ compared to the neat epoxy homopolymer. A similar strategy, albeit one based on simple monomers, was adopted to combine a photochemical thiol-ene reaction with a photoinitiated cationic homopolymerization of epoxides [204,205]. This curing system made it possible to prepare crosslinked materials with $T_{g}$s that depended on the formulation composition, being higher when the amount of epoxide increases.

As a one-pot approach, Jin et al. described a thiol-acrylate-epoxy system wherein a nucleophile catalyzed thiol-Michael addition took places concurrently with a base (1,8-diazabicyclo[5.4.0]undec-7-ene, DBU) catalyzed thiol-epoxy reaction [206]. By using high molecular weight acrylates in the formulation, phase-separated thermosets were obtained. The researchers defined their system as a hybrid polymer network (HPN) with versatile thermal and mechanical properties. Depending on the functionality of the employed thiol, materials with Young’s moduli ranging from 1.5 to 75.7 MPa were obtained. By changing the cross-linking density in this way, the extent of phase separation was also controllable, which, in turn, dictated the macroscopic properties of their materials. Sequentially-cured thiol-acrylate-epoxy ternary systems were also documented, with stable and tailorable intermediate materials [27,207]. In the former work, the different reaction rates facilitated the sequentiality of the process, where a click thiol-Michael reaction was carried out at room temperature, followed by thiol-epoxy curing at elevated temperature. As catalyst, the researchers tested 4-(*N*,*N*-dimethylamino)pyridine (DMAP), and 1-methylimidazole (1-MI), and saw that DMAP provided better separation between the two curing stages. In the latter work, the sequentiality of the curing was achieved by employing the same TBD·HBPh_4_ photobase generator shown in Scheme 15. To maintain the stoichiometric ratio of thiol:acrylate:epoxy at 2:1:1, the authors resorted to using a radical scavenger to inhibit radical-mediated reactions during UV irradiation such as acrylate homopolymerization. As expected, the addition of a thiol-epoxy step as a secondary curing stage helped increase the T_g_ of the final materials significantly.

As mentioned in Section 3, epoxides are most easily cured by amines. It is therefore not uncommon to encounter an epoxy-amine reaction in some dual-click procedures. One such system was reported by Murakami et al. in the area of hydrogels, wherein an epoxy-amine reaction was combined with a copper-catalyzed azide-alkyne cycloaddition in a modular one-pot system [208]. The motivation to design such a dual-curing process stems from the complexity of dual-network gel preparation and inherent problems such as swelling and brittleness. Since each network contributes differently to the density of crosslinks, a durable material with tightly controlled properties could be obtained simply by changing network weight fractions.

Dual-curable epoxy thermosets can be obtained completely in a “click manner” by using a combination of aliphatic and aromatic diamines. By employing these two amines which are reactive at two different temperatures, an epoxide compound (triglycidyl p-aminophenol epoxy resin) was dual-cured without any catalysts [209]. Depending on the initial monomer composition, partially-cured (stage 1) materials were either liquid or gel, each of which could be used for specific applications. Liquid materials were successfully tested as adhesives and gels were shape-conformable, with the possibility of fixing the shape permanently by triggering and carrying out the second curing stage. The two-fold intermediate stability, thanks to both the low reactivity of the aromatic amine at storage temperatures and to the vitrification of the partially-cured network, was elegantly represented by a time-temperature-transformation diagram in a subsequent publication [49].

## 10. Advanced Technological Applications of Thermosets Obtained by Means of Click Methodologies

As mentioned in previous sections, the applications of click chemistry span a broad range of technological fields from optical devices, printing materials (3D printing) and microfluidics to recently emerged shape memory, self-healing or remoldable polymers, also encompassing conventional adhesive materials or coatings.

A prominent application of click chemistry, namely thiol-ene/thiol-Michael chemistry, is in the field of optical materials. A thorough review from Du Prez et al. [102] highlights the application of thiol-ene and thiol-Michael reactions for surface modification and coatings in optical materials. Transparent optical polymers are essential in numerous applications thanks to their low cost, lightness and ease of processability compared to traditional glass. Materials obtained by this chemistry are able to meet the main requirements of high refractive indexes and low chromatic dispersion (or high Abbe number) for use in specific optical applications. If adequately modified, they also meet specific mechanical performance requirements, i.e., scratch resistance, hardness and flexibility. One such modification is the synthesis of highly branched poly(thiourethane) acrylates and their use in a thiol-Michael reaction to obtain materials with good mechanical properties while maintaining high refractive indexes (up to 1.62) [210]. Other strategies have focused on the incorporation of inorganic components like silsesquioxanes. Gan et al. [211] obtained a photo-curable silicone liquid resin based on thiol-ene photopolymerization with a refractive index of around 1.54 and optimal mechanical properties for the encapsulation of LED chips.

As stated in Section 6, crosslinked poly(thiourethane)s have also been widely used in optical materials thanks to their high refractive indexes [126,127]. The materials described by Jia at al [126] based on commercially available diisocyanates and tri- and tetra- thiols of different structures and prepared using dibutyltin dichloride as the catalyst exhibit high refractive indexes (around 1.66) and high tensile strength, thereby meeting optical lens requirements.

Excellent materials for optical applications were obtained also by Bowman and coworkers [191]. They synthesized thiol-isocyanate-acrylate ternary networks by the combination of thiol-isocyanate coupling, thiol-acrylate Michael addition and acrylate homopolymerization. The obtained materials presented a refractive index of about 1.55, making them suitable for use as optical lenses. A similar refractive index [190] was obtained with thiol-isocyanate-ene ternary networks prepared by sequential and simultaneous thiol-ene and thiol-isocyanate click reactions.

Another interesting market where click chemistry can fit perfectly is flexible bioelectronics. Here, there is an ever-growing need for high performance materials. In the case of thiol-click reactions involving epoxide, alkene, or (meth)acrylate reactive groups, the resultant thioether linkage demonstrates an inherent flexibility that yields a significant reduction in the cure-stress of the final network, a quality that is desirable for low-shrinkage uses, such as the photolithographic fabrication of flexible electronics [3]. However, the low moduli of these materials due to the thioether linkage have sometimes led to substrate failure due to insufficient mechanical rigidity for penetrating soft tissue at physiological temperatures, or insufficient softening capacity to reduce the mechanical mismatch between soft tissue and the implantable device. Voit’s research group successfully explored the ternary thiol–epoxy/maleimide network as potential substrate materials in which the degree of softening can be modulated without sacrificing the mechanical rigidity at physiological temperatures [89,212,213]. With the same aim of obtaining specific thin films for sensing devices and exploiting the shape memory properties of thiol-acrylate systems, they also fabricated a flexible polymer substrate with enhanced dimensional stability and adhesion that can be used as a platform for the development of flexible bioelectronics by photolithographic processes [214].

Obtaining lithographic printing materials is another field where click chemistry has been applied with success. In the work presented by Bowman’s group [215], thiol-ene polymerization was demonstrated to be an effective method for step and flash imprint lithography thanks to rapid polymerization rates, minimal oxygen inhibition, near-complete polymerization and lower shrinkage compared to the acrylate polymerization. The strategy developed by Bowman’s group is based on the formation of functionalized nanopatterned substrates with attached secondary ultrathin linear and crosslinked polymer films which facilitate the control of topology and chemistry of the nanopatterned surface in a convenient manner. The step growth mechanism of the thiol-ene polymerization facilitated the control of the polymer properties by changing the stoichiometry and functionality of the monomers.

Also based on thiol-ene chemistry, Bowman’s group presented remoldable vitrimers for photopatterning and nanoimprint lithography [216]. These materials combined the groundbreaking properties of advanced materials, i.e., reshapability, remoldability and shape memory at the nanoscale. An interesting review on nanoimprint lithography presented by this research group summarizes the emergent materials used, most of which were based on click chemistry [217].

Following a dual-curing approach as presented in Section 9, the Bowman’s group also came up with an elegant process for preparing holograms [194]. They first prepared loosely cross-linked soft elastomeric substrates through a thiol-acrylate Michael addition. After the first stage, the intermediate materials contained uncured -ene and excess thiol groups which underwent UV-initiated click thiol-ene photopolymerization, allowing holographic images to be recorded onto them. The final materials were graded rainbow holograms with high performance. Also using a dual-curing system (a base catalyzed thiol-Michael addition followed by a radical-initiated thiol-yne click reaction), they presented an image patterning technique for applications such as high-density data storage, image patterning and anti-counterfeiting [196].

Another innovative application of thiol-ene click-chemistry is in microfluidics. Although there is not a “perfect” material that fits all the requirements in microfluidics, the thiol-ene family has enormous potential for organs-on-a-chip applications, droplet productions, microanalytics and point of care testing. The fabrication of microfluidic devices from such materials is typically done by the classical photolithography, replica molding (or double replica molding) or reaction injection molding, and allows very fast prototyping to be performed. This possibility, combined with good mechanical strength, solvent resistance, biocompatibility and the inherently easy surface functionalization are the distinctive features that make thiol-ene polymers suitable for microfluidic applications. Another advantage of thiol-ene polymers for obtaining microfluidic devices with complex shapes is that they can bond using different strategies (semicuring, off-stoichiometry and dual-curing with ternary materials). In their review, Carlborg and coworkers demonstrated how off-stoichiometric thiol-ene processes are suitable for the commercial production of microfluidic devices to substitute classical poly(dimethylsiloxane) (PDMS). The polymers used were based on versatile UV-curable thiol-ene chemistry, taking advantage of off-stoichiometric ratios to offer important features for a prototyping system, such as one-step surface modifications, tunable mechanical properties and leakage-free sealing through direct UV-bonding [218]. In a more recent review by Kutter et al. [219], the main characteristics of the thiol-ene materials and their use in microfluidic devices were presented critically by thoroughly analyzing the advantages and drawbacks.

**Figure 8:**
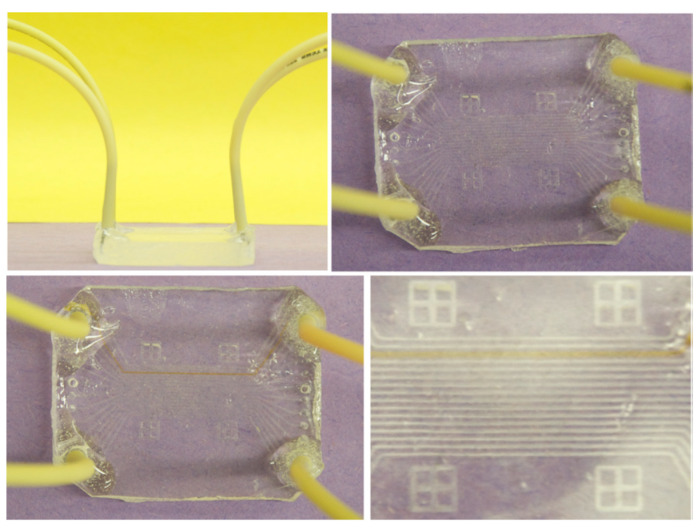
Images of the final annealed microfluidic device attached to inlet capillaries (top left and top right) and methyl orange solution being pumped through final microfluidic device. Reproduced with permission from [220]. Copyright 2020, Elsevier Ltd.

Stable and tunable hydrophilic-surfaced materials for microfluidic applications were also obtained by Pojman et al. [220] via the in situ, tertiary-amine-catalyzed Michael addition of a multifunctional thiol to a multifunctional acrylate. As shown in Figure 8, a complete microfluidic device was obtained, demonstrating the potential of these systems in microfluidics.

Khan and Yeo created soft micro- and nano- patterned surfaces based on the thiol-epoxy click reaction [221] catalyzed using a photobase generator and employing different soft lithography techniques. The network structure and bulk properties of the materials could be easily tailored by suitable combinations of thiol and epoxy monomers, which would yield patterned surfaces with enhanced resolution. Postmodification was possible by the alkylation of the thio-ether bonds formed in the thiol-epoxy reaction, making it possible to add diverse functionalities such as fluorescence or anti-fouling [221]. Similar modifications were also reported for hydrogels prepared by thiol-epoxy click reaction in the presence of water [222]. Surface functionalization by the alkylation of thio-ether bonds is a versatile approach that could also be extended to other materials based on thiol-click chemistries such as thiol-ene or thiol-yne.

**Figure 9:**
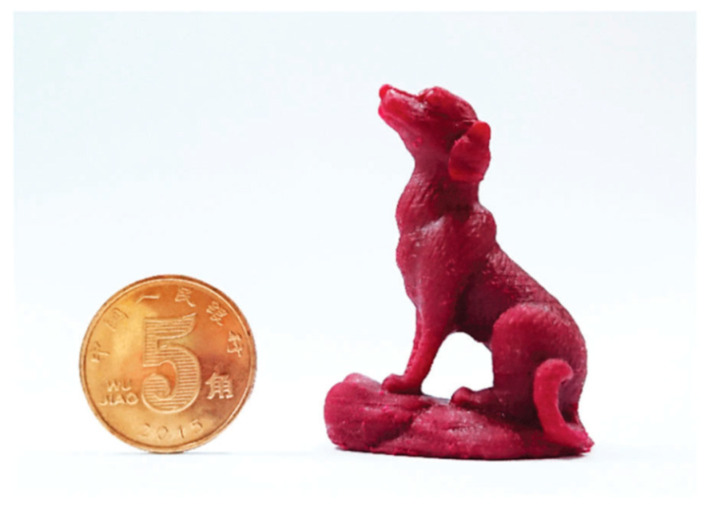
Digital picture of secondary thiol-ene (PE-1/TTT) photosensitive resin after DLP printing. Reproduced from [223] with permission from the Royal Society of Chemistry.

Thanks to their high refractive index, low oxygen inhibition and minimal polymerization-induced shrinkage, click thiol-enes are well-suited for use as 3D printing resins. In the work presented by Chen et al. [223], digital light processing 3D printing is applied to fabricate objects with high vertical resolution and high refractive index (>1.54) using thiol-ene systems with tunable mechanical properties. An example of this can be seen in Figure 9.

Thiol-ene ‘click’ reactions utilizing terpenes and a four-arm thiol have also been employed to produce thermoset 3D-printed structures using vat photopolymerization [104]. The obtained materials showed a broad range of thermomechanical properties thanks to changes in their composition and the thermal curing profiles used. Qualitatively, linalool was the most effective monomer among the terpenes used for printing (limonene, nerol and geraniol) due the combination of its low viscosity and its reactivity. The limonene monomer was only able to produce 2D designs without complexity in the z-axis, and nerol and geraniol were could successfully be printed into 3D structures as a consequence of their slow photo-crosslinking behavior.

**Figure 10:**
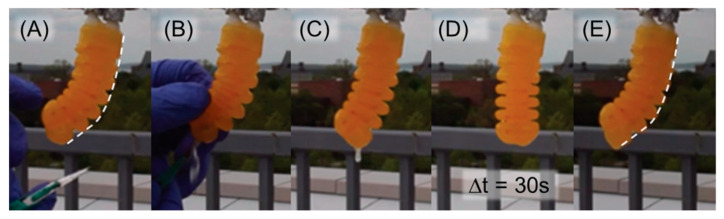
Synthetic antagonistic muscle actuator printed from the 2.5% 186 resin: (**A**) pressurized with low-viscosity prepolymer resin; (**B**) pierced by a scalpel; (**C**) pressurized fluid draining; (**D**) autonomous self-healing via ambient sunlight in the relaxed state for 30 s (**E**) returning to its original actuated state (dashed line) with repressurization. Reproduced from [224] with permission from the Royal Society of Chemistry.

Shepherd’s group also used thiol-ene click chemistry wisely to obtain tunable materials for soft robotics fabricated via stereolithography [224]. Their materials can be easily tuned to achieve storage moduli ranging from 6 to 283 kPa through the appropriate selection of the two primary chemical constituents (mercaptosiloxane and vinylsiloxane). Moreover, the fabricated materials showed excellent resilience, a property required for a soft machine that undergoes many actuation cycles. The high strain and resilience, and low elastic modulus of these materials are similar to biological tissues, making them ideal for manufacturing soft robots. To demonstrate the applicability of the materials in soft robotics, they fabricated an antagonistic pair of fluidic elastomer actuators (a primary component in most soft robots) that can self-heal via sunlight (see Figure 10).

The self-healing ability, a topic of scientific and technological interest at present, is another outstanding property of some thermosets obtained by click chemistry. Some examples have already been presented in previous sections. The self-healing materials presented in [69] are based on the fast thiol-epoxy reaction in the presence of tertiary amines and the addition of two types of microcapsules, a thiol/amine mixture and another with epoxy resins. When these capsules break, they release both components that react and heal the crack instantaneously.

Another example, already mentioned in Section 6, is from Du Prez’s research group [125]. In their work, the fast thiol-isocyanate chemistry, combined with a dual capsule strategy (with isocyanates and thiols), was used for the development of extrinsic self-healing epoxy materials. Using a similar strategy of encapsulated reagents for self-healing, Binder’s research group described the successful encapsulation of liquid reactive components for the azide/-yne “click”-reaction [225]. Additionally, Siddaramaiah et al. [226] described a self-healing material based on dual component microcapsules of epoxy-resin and a hardener embedded in epoxy matrix.

Besides the extrinsic self-healing technique, reversible bonds are also attractive for obtaining a self-healable material. In a work presented by Zhang et al. [227], smart poly(caprolactone) (PCL) networks were obtained by blending a disulfide monomer and a branched PCL cured with thiols via a thiol-ene reaction. The materials obtained were able to repair mechanical damages efficiently, and they also present shape memory, reprocessability and degradability. Similarly, the Konkolewicz’s research group [228] developed a new dynamic polymer system based on thermally responsible thiol-Michael adducts which are healable and malleable and have long-term mechanical stability in the absence of stimulus. Albeit without self-healing ability, but with other smart properties like shape memory or reshaping, Miao et al. [229] recently reported the synthesis of PCL networks with adjusted crosslinking densities and hydroxyl contents using a thiol-ene click reaction. The click nature of the thiol-ene reactions make it possible to precisely control two network parameters: crosslinking density and hydroxyl content. This allowed them to obtain thermally adaptable shape memory polymers with tunable performance. The reader is directed to the paper by Du Prez et al. [230] for an excellent review of the chemistries for autonomous external self-healing polymers and composites.

Finally, a cutting-edge application of click-based thermosets is Covalent Adaptable Networks (CANs). These constitute a novel material class with the possibility of being reshaped, remolded and recycled, with shape-memory properties and/or self-healing abilities. There are excellent papers in the literature presenting different systems or properties of CANs, of which some have already been cited in the text. However, to summarize them exhaustively is beyond the scope of this paper. Excellent reviews were published by Bowman’s group [167,231,232], Du Prez’s group [233] and, more recently, by Jin et al. [234] and Scheutz et al. [235]; the reader is encouraged to consult these publications for more in-depth information.

Given the aforementioned applications, without intending to be exhaustive, this last section has tried to convince the reader that click chemistry opens up a huge world of smart, intriguing and fascinating applications that are (predominantly) technologically available. Being simple, environmentally-friendly and therefore sustainable, click procedures might fully replace conventional thermoset fabrication techniques in the near future to become the go-to approach not only for basic industrial applications, but also complex and delicate, high value-added technological applications. It is the belief of the authors that, with the interdisciplinary efforts that this review will hopefully inspire, a lot of ground can be covered in this promising field rooted in organic chemistry, but rootlets touching many other disciplines such as polymer science and material engineering.

## 11. Conclusions

Click chemistry is a versatile tool providing extensive control in the preparation of thermosets with tailored structures and properties. The smooth reaction conditions, the high yields achieved and the possibility of performing most of these reactions in air, and even in humid environments, make them ideal for industrial use. In addition, the power of their orthogonality makes it possible to establish novel advanced fabrication procedures.

Considering the ever-growing number of applications of click-based thermosets, some of which were outlined in this review, we expect that these efficient synthetic methodologies will be more widely implemented to tackle challenging problems in industry. We are convinced that the application of click chemistry will contribute to improving industrial productivity, mitigating environmental impact and opening up new possibilities in emerging technologies.
